# A Comprehensive Review of Cross-Linked Gels as Vehicles for Drug Delivery to Treat Central Nervous System Disorders

**DOI:** 10.3390/gels8090563

**Published:** 2022-09-06

**Authors:** Leshasha T. Mashabela, Mahlako M. Maboa, Ntombi F. Miya, Taiwo O. Ajayi, Rumbidzai S. Chasara, Marnus Milne, Shoeshoe Mokhele, Patrick H. Demana, Bwalya A. Witika, Xavier Siwe-Noundou, Madan S. Poka

**Affiliations:** Department of Pharmaceutical Sciences, School of Pharmacy, Sefako Makgatho Health Sciences University, Pretoria 0204, South Africa

**Keywords:** cross-linked gels, central nervous system, spatial drug delivery, injectable cross-linked gels, non-injectable cross-linked gels

## Abstract

Gels are attractive candidates for drug delivery because they are easily producible while offering sustained and/or controlled drug release through various mechanisms by releasing the therapeutic agent at the site of action or absorption. Gels can be classified based on various characteristics including the nature of solvents used during preparation and the method of cross-linking. The development of novel gel systems for local or systemic drug delivery in a sustained, controlled, and targetable manner has been at the epitome of recent advances in drug delivery systems. Cross-linked gels can be modified by altering their polymer composition and content for pharmaceutical and biomedical applications. These modifications have resulted in the development of stimuli-responsive and functionalized dosage forms that offer many advantages for effective dosing of drugs for Central Nervous System (CNS) conditions. In this review, the literature concerning recent advances in cross-linked gels for drug delivery to the CNS are explored. Injectable and non-injectable formulations intended for the treatment of diseases of the CNS together with the impact of recent advances in cross-linked gels on studies involving CNS drug delivery are discussed.

## 1. Introduction

Central nervous system (CNS) disorders represent an area of major unmet medical need [1]. They are highly prevalent, devastating, and yet poorly treated illnesses [2]. Common neurodegenerative diseases such as Alzheimer’s disease (AD), Parkinson’s disease (PD), and amyotrophic lateral sclerosis (ALS) are associated with increasing age and genetic predisposition [3]. The prevalence of neurodegenerative diseases presents a major threat to the health of the elderly population. Parkinson’s disease, for example, causes disabling symptoms such as tremors, muscle stiffness, and postural instability, which can cause falls resulting in further debilitation [4,5]. Alzheimer’s disease, on the other hand, causes memory and cognitive impairments, while ALS affects basic motor neuron functions such as speaking, swallowing, and breathing [3,6]. Chronic CNS disorders such as epilepsy and schizophrenia require long-term drug therapy to control symptoms [5,7].

The treatment of these neurological disorders is mainly hindered by the blood-brain barrier (BBB), whose physiological function is to regulate the transport of substances to the brain and prevent the entry of foreign substances that could damage it [8], resulting in inadequate drug concentrations reaching neuronal tissues [5]. In the clinical setting, attempts to circumvent BBB effects on CNS drugs often include elevation of doses or frequent dosing intervals. However, this may result in high plasma levels of these drugs increasing their potential to cause serious adverse drug events. As such the development of novel drug delivery systems for local or systemic drug delivery of CNS drugs in a sustained, controlled, and targetable manner have been at the epitome of recent advances in drug delivery systems. In recent years, advances in drug delivery systems have been focused on effective, easily produced, and reproducible techniques. Gels have attracted the attention of researchers in innovative drug delivery techniques owing to their ease of production, tampering, and their ability to elicit controlled or sustained drug release patterns locally at sites of absorption or action.

Gels can be classified based on various characteristics including the type of solvent used during preparation and its physical nature [9]. Cross-linked gels are a class of gels that are based on cross-linked polymeric systems. They are formed by physical or chemical cross-linking of polymeric chains, generating swellable polymer networks [10]. The polymer used can be either natural, synthetic, or in some cases, a combination of both. The wide variety of polymer choices and versatile architecture of cross-linked gels allow them to incorporate a plethora of both hydrophilic and lipophilic molecules, ranging from inorganic molecules to biomolecules such as nucleic acids, amino acids, and proteins without losing their gel-like behavior [11,12].

Cross-linked gels can be modified by altering their polymer composition and content for pharmaceutical and biomedical applications. These modifications have resulted in the development of stimuli-responsive dosage forms that offer many advantages with regards to the effective dosing of CNS-acting drugs.

In this review, applications of cross-linked gels as drug delivery systems are discussed while also highlighting their application as both injectable and non-injectable formulations intended for the treatment of diseases of the CNS.

## 2. Challenges in CNS Drug Delivery

Over the years, advances in CNS drug delivery have led to increased research output concerning safe and effective drug delivery systems that could improve drug penetration into the CNS. The CNS parenchyma is a highly specialized tissue protected by a blood-brain barrier (BBB). The BBB is a highly selective membrane located between the blood and brain interstitium [13]. It regulates molecular exchange pathways that transport molecules from blood to brain and brain to blood [13]. It separates peripheral circulation from the CNS, thereby protecting the brain from unwanted molecules and foreign substances allowing only the exchange of small molecules that are permeable to the BBB into the brain by diffusion [5,14]. Unfortunately, the BBB along with the presence of the blood-cerebrospinal fluid barrier and efflux transporters presents a huge challenge for the effective delivery of therapeutics to the CNS [14,15]. Many effective therapeutics have failed to cross these barriers and have been discarded during their development for clinical use due to a failure to reach the CNS in sufficient quantity [14].

### 2.1. The Blood-Brain Barrier (BBB)

**Figure 1:**
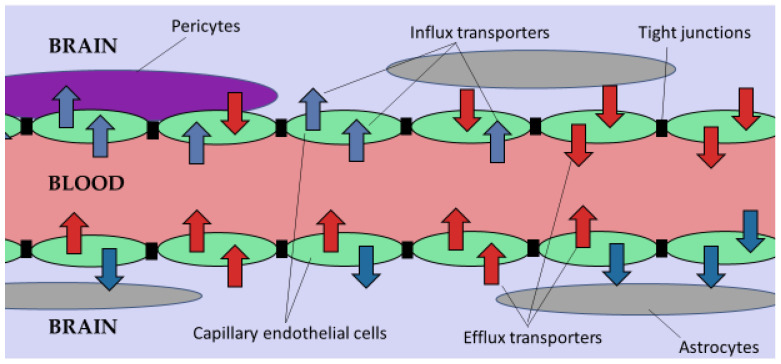
The blood-brain barrier and drug transporters in the capillary endothelial cells. (Blue arrows: Flow of molecules through influx transporters into the brain parenchyma from the blood vessel; Red arrows: Outward flow of molecules through efflux transporters from the brain parenchyma to the blood vessel) [Adapted from [17] in terms of the Creative Commons Attribution License (CC BY 3.0)].

The blood-brain barrier (BBB) is a complex and unique semi-permeable membrane that protects the brain to maintain homeostasis within it [16]. It inhibits the entry of molecules greater than 400 Daltons from the bloodstream into the brain, thereby protecting the brain microenvironment from potentially harmful exogenous molecules [16]. The BBB selectively permeates passive transport of substances such as water, nutrients, and hydrophobic, while its protective mechanism involves active efflux of lipid-soluble toxins and bacteria and is mediated by P-glycoprotein (P-gp) [5]. As depicted in Figure 1, the BBB cerebrovasculature is composed of a specialized endothelial cell monolayer supported by pericytes and astrocytes creating tight interconnectivity and a unique barrier. Vascular pericytes surround 30% of the endothelial layer, controlling the vessels’ diameter and regulating endothelial cell BBB-specific gene expression [16]. Perivascular astrocytic end-feet, attached to the external wall of the endothelium, is critical for the proper functioning of the BBB and strengthens the tight junctions [5]. The tight junctions between adjacent vascular endothelial cells restricts paracellular movement of molecules, favoring the transcellular pathway which involves transporter proteins and the BCSFB.

### 2.2. The Blood-Brain Cerebrospinal Fluid Barrier (BCSFB)

While the BBB is considered to be localized within CNS microvessels at the endothelial cells level, the BCSFB is formed by epithelial cells of the choroid plexuses (CPs) located in the four ventricles of the brain and the subarachnoid epithelial structures facing the CSF spaces in the intracranial and spinal areas [18]. The CP inhibits paracellular diffusion of polar molecules across the barrier in addition to producing and secreting cerebrospinal fluid (CSF) [19]. The BCSFB is strengthened by the expression of active organic acid transport systems which allow for the direct transport of CSF-borne organic molecules into the blood, subsequently removing several drugs out of the CSF, inhibiting them from crossing into the brain parenchyma [5].

### 2.3. Efflux Transporters

In addition to shielding the brain parenchyma, the BBB also serves as a transport regulator for passage of molecules to and from the brain. Transport into the brain occurs by either paracellular transport or transcellular transport [14]. Paracellular transport involves passage between endothelial cells, while transcellular transport involves passage through the cell from the luminal to the abluminal surface of the endothelial cell into the parenchyma of the brain [14]. Multi-specific efflux transporters belonging to the ATP-binding cassette (ABC) transporter superfamily are differentially localized between the two main blood-brain interfaces, the BBB and BCSFB [20]. The two prominent efflux transporters are P-glycoprotein (Pgp) and breast cancer resistance protein (BCRP) [5,20,21]. They use ATP hydrolysis to translocate both endogenous and exogenous compounds, including drugs, from the brain into the blood against their concentration gradient [5,13].

P-glycoproteins (Pgp) is highly expressed at the BBB [20]. It is encoded by the multidrug resistance gene MDR1 and is the main drug efflux transporter of the BBB. It is responsible for many drug-resistant CNS disorders including brain tumors. This was first reported in 1995 by Tishler et al., who linked increased Pgp expression to medically intractable epilepsy [22,23]. Similarly to Pgp, BCRP is expressed at the BBB. Studies have demonstrated the role of BCRP in cancers as stem cell differentiation, protection against xenobiotics, and cancer cell survival under hypoxic conditions as there is increased expression of the transporter in tumor stem cells. However, in brain tumors, this is not always the case. For example, in primary CNS lymphoma, there is down-regulated expression of the BCRP transporter [24], while it is highly up-regulated in neuroepithelial tumors such as ependymomas, causing multidrug resistance [21,25]. Drugs and other substances that escape the ABC transporters are pumped out of the brain parenchyma via a second mechanism, called phase I and II metabolism [5]. The phase I and II metabolism reduces the lipid solubility of drug molecules by (I) either uncovering or adding reactive polar groups to the surface of a drug molecule with enzymes such as cytochrome p450, or by (II) conjugation of anionic groups onto the drug molecule by transferases such as glutathione S-transferase, rendering the molecule ready for expulsion and ineligible for re-entry [5].

### 2.4. Effects of CNS Diseases on the BBB and Drug Delivery

Drug penetration through the BBB and distribution within the brain CSF is governed by many factors, including physicochemical and pharmacokinetics properties of the drug, blood perfusion in the brain capillaries and BBB integrity [26]. All of these factors may be affected by different CNS diseases. For example, a study conducted on patients with Huntington’s disease (HD) demonstrated a reduction in the integrity of the BBB and considerable disruption of the cerebral vasculature following magnetic resonance imaging as well as post-mortem tissue analyses [27]. In brain tumors, the BBB may be disrupted by the dramatic vasculature changes during tumor progression; the proliferating cancer cells have nutritional demands that may require co-opting existing vessels and/or creating new ones via angiogenesis [28]. The angiogenesis process is in part mediated by the expression of angiogenic factors such as vascular endothelial growth factor (VEGF) [28], which is also increased during brain injury [29]. However, VEGF increases the permeability of the BBB via the synthesis and release of nitric oxide, which causes dilatation of arteriolar vessels and increases vascular permeability to enhance increased tumor blood flow [30]. The disruption of the BBB may result in higher drug accumulation within brain tumors [28,30].

In various CNS diseases, the expression of efflux transporters is enhanced, leading to a reduction in drug uptake by the brain. One example of drugs affected is riluzole. Riluzole is a drug used in ALS treatment but is not very efficacious as it is a substrate for P-gp and BCRP [31]. The efflux transporters P-gp and BCRP are both upregulated in ALS, making it more difficult for therapeutic agents to cross the BBB [32]. Several strategies targeting efflux transporters, angiogenesis factors, and other factors threatening the integrity of the BBB have been explored to improve the delivery of drugs into the brain [32]. Other degenerative CNS disorders such as AD, PD cause BBB dysfunction with simultaneous increase in gliosis, neurovascular dysfunction, neuroinflammation and progressive loss of nerve function [33]. Chronic psychiatric disorders including schizophrenia also compromise neurovascular health [33].

## 3. Classifications of Cross-Linked Gels

**Figure 2:**
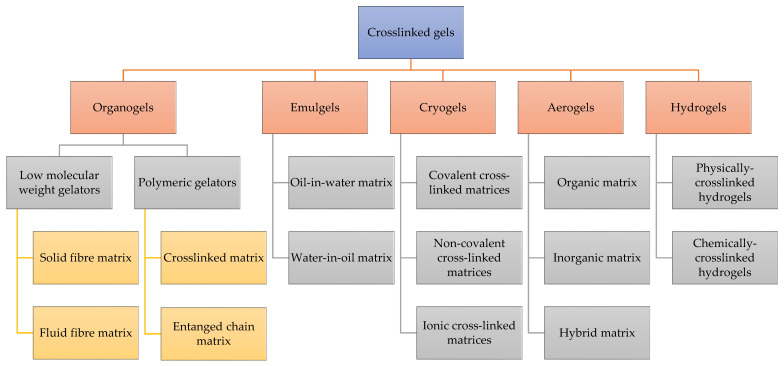
Classifications of cross-linked gels based on the nature of solvents.

Cross-linked gels can be classified into various categories based on the source, structure, cross-linking method, charge, and biodegradability. One of the most commonly used classifications is based in the nature of solvent in the continuous phase and is depicted in Figure 2.

### 3.1. Emulgels

Emulgels, a portmanteau of emulsions and gels, are an integration of gels and emulsions. Their use has emerged both in cosmetic and pharmaceutical preparations [34]. Emulgels offer enhanced stability as they possesses characteristics of both gels and emulsions [35]. They operate as dual-control drug release systems [34]. They are also emerging as a drug delivery system for hydrophobic drugs. With the aid of a gelling agent, two types of emulgels can be formed viz., oil in water (o/w) and water in oil (w/o) [36]. There are various properties of emulgels that enable them to provide better control of drug release in topical application such as thixotropicity, ease of spreadability, long shelf-life, improved loading efficiency, and enhanced stability [36,37]. Some of the limitations associated with emulgels are that they pose a challenge with absorption of macromolecules and during formulation as they are prone to entrap air bubbles [38]. O/W emulgels are widely used in general cosmetic preparations because of their washable nature while for skin ailments water-in-oil emulgels which are emollient are used [37]. Constituents that are used in the formulation of an emulgel include a vehicle, aqueous material, oil, emulsifiers, gelling agent, penetration enhancers, pH adjusting agent, and an active pharmaceutical ingredient (API) [39]. The three general steps involved in the preparation of an emulgel are, firstly, the formulation of o/w or w/o emulsion followed by the formulation of a gel base, and then the emulsion is incorporated into a gel base with continuous stirring [40]. Lampp et al. prepared emulgels that remained stable in-vivo for up to 3 months following subcutaneous injection with no accompanying irritation or toxicity [41]. The emulgel was based on isopropyl myristate and 12-hydroxystearic acid as gelling agent to deliver molecular probes for prolonged, localized measurements of molecular oxygen [41]. The stability reported indicates that encapsulation of drugs within the emulgel matrix could achieve prolonged drug residence time at the site of injection. This is a particularly impressive feature for potential applications of emulgels in the CNS.

### 3.2. Organogels

An organogel is a class of semi-solid preparation that is composed of an organic liquid phase in an immobilized three-dimensional cross-linked network. Despite its liquid composition, this viscoelastic system takes on the same characteristics as a solid [42]. Organogels have an external apolar phase that gets immobilized within the three-dimensional structure formed due to the physical interactions amongst gelators which are the self-assembled structures of compounds [43]. Organogels can be used for drug delivery via routes such as transdermal, oral, and parenteral. The use of organogels as drug delivery systems is limited by the lack of biocompatibility for these formulations. However, the development of biodegradable and biocompatible organic solvents and organogelators has made them more pharmaceutically friendly [44]. According to the nature of the gelator, organogels are classified as low molecular weight gelators and polymeric gelators. Depending on the nature of intermolecular interactions, low molecular weight gelators can be physically categorized as solid-fiber matrix and fluid-fiber matrix. Polymeric gelators can be physically classified as entangled-chain matrices and chemically classified as a cross-linked matrices [45]. Wang et al. prepared and characterized a biodegradable in- situ forming organogel, obtained through the self-assembly of long chain fatty acid in pharmaceutical oil [46]. Generally, organogels are easily formulated by dissolving an organogelator into a hot, apolar phase followed by a cooling step which results in gelation [44]. Organogels have a good list of merits that make them ideal drug delivery systems including controlled release of drugs, good stability, prolonged action, reduced dosing frequency, and enhanced drug penetration through the skin [43]. While the history of organic solvent toxicity limits the application of organogels in biomedical applications, more recent studies synthesize organogels with better biocompatibility.

### 3.3. Cryogels

Cryogels are formed by the subsequent melting of ice crystals, leaving behind a polymeric material with an interconnected macroporous network surrounded by dense polymer walls [47]. At subzero temperatures (typically between −5 and −20 °C), cryogels are formed via a process called cryogelation [48]. Cryogels possess a unique set of properties including substantial pore size and porosity, high water content, great pore connectivity, and consistency. These properties contribute to physical resilience, bio-adaptability and facilitates cellular migration, tissue-ingrowth, and diffusion of solutes, including nano- and micro-particle trafficking, into its supermacropores. The sponge-like microporous system allows for a more rapid swelling kinetic profile and improved viscoelastic properties preventing physical deformation [49]. Similarly to conventional gels, cryogels can be divided into three categories based on the type of interchain linkages present in the network’s junction knots: covalent (cross-linked chemically), non-covalent (physical), and ionically cross-linked matrices [50]. Their merits include the flexibility of their preparation and operating mostly with water as a solvent, making them more economical and environmentally friendly [47]. Cryogel scaffolds can be readily formed through physical cross-linking, including ionic interactions and hydrogen bonding. Poly (vinyl alcohol) (PVA) is one of the most studied synthetic polymers that can be physically crosslinked to form cryogels [51]. Chemical cross-linking can provide satisfactory tailored mechanical properties, but toxic compounds are used as cross-linkers, are difficult to extract, and can affect biocompatibility.

Stimuli-responsive properties are frequently desirable for biomaterials employed in drug delivery applications. A temperature-responsive polymer experiences phase shifts, which physically alter its shape and/or characteristics at certain and distinct temperatures. As the solubility of a polymer in a solvent, or solvation state, changes with temperature in thermally responsive cryogels, it is frequently described in terms of swelling [52]. Dual shape memory behavior in thermoresponsive cryogels containing oligoethylene glycol has been developed [53]. A number of dendronized IPN hydrogels utilizing the cryogelation approach were created using covalently crosslinked polyacrylamide for one network and reversible Schiff-base crosslinked oligoethylene glycol (OEG)-based dendronized polymers for another network. Along with having an interconnected porous architecture and excellent mechanical strength, these cryogels also inherit OEG-based dendronized polymers’ distinctive thermoresponsive properties and display thermally induced deswelling behavior when heated over their phase transition temperatures [53]. The cryogel’s chemical makeup and the characteristics of the medium, such as its pH, ionic strength, and medium composition, all influence the degree of swelling [54]. This has an impact on the interactions of pH-sensitive polymers (both polymer-solvent and polymer-polymer interactions), as functional groups on the polymer chains might exhibit weak acidic or basic properties depending on how protons react to pH variations [55]. Cryogels made of poly(acrylic acid) have been studied as prospective soft materials for energy production as a pH oscillator in oscillatory bromate-sulphite-ferrocyanide processes [56]. By combining acrylic acid monomer and *N*,*N*′-methylenebis(acrylamide) crosslinker at 18 °C in water, macroporous poly(acrylic acid) (PAAc) cryogels were created. The reactions were run at several initial monomer concentrations to determine the ideal setting for the synthesis of quickly responsive PAAc gels, with the help of the bromate oscillator, PAAc cryogels oscillate between swollen and collapsed states, with each cycle resulting in a three-fold increase in the gel volume [56].

### 3.4. Aerogels

The quick rise of aerogels in the field of drug administration is attributed to their composition diversity, modularity, and viability of industrial scale fabrication [57]. By definition, an aerogel is created when a gel’s liquid content is swapped out for gas. Aerogels as drug delivery matrices for a variety of administration routes (e.g., oral, pulmonary, nasal, topical) are able to enhance the therapeutic result with changed drug release profiles in pharmaceutical applications. Aerogel-based therapeutic systems can manage the delivery of bioactive molecules and greatly increase medication bioavailability [58]. In a nutshell, the construction of aerogels starts with the preparation of either ahydrogels or organogel. A “sol” of precursors, water, and a catalyst is used to create the gel. Typically, this is achieved by adding a chemical cross-linker or by altering the physical conditions of the reaction (e.g., pH, temperature). Washing and/or solvent exchange with a suitable solvent are the next stages [59]. In general, aerogels’ physical characteristics include their solid state’s exceptionally low density (0.0001 to 0.2 g/cm^3^), high specific surface area (>200 m^2^/g), and porosity of at least 90% with a majority of open pores in the mesoscale (2–50 nm). Additionally, this unique class combines exceptional textural features, and, in most cases, tunable surface chemistry [60].

A number of parenteral and particularly mucosal administration routes for drug delivery seem to benefit greatly from several physical features of aerogels. Aerogels’ strong capacity to absorb liquids may make it easier to precisely control the absorption of exudates from skin wounds and maintain moisture levels that aid in wound healing while the aerogel still controls medication release [57]. Aerogels face difficulties such as low mechanical strength in some instances and the economic and environmental costs of the supercritical drying technique used to complete the synthesis process. Aerogels can generally be divided into three categories: organic, inorganic, or hybrid (based on their composition), and microporous, mesoporous, or mixed (based on the size of their pores) [61]. The ability of alginate aerogels to encapsulate tiny pharmaceuticals with improved controlled release was demonstrated by the incorporation of medications such as niacin/nicotinic acid and ibuprofen into the aerogel’s architecture at various stages of the production process [61].

### 3.5. Hydrogels

Hydrogels are three-dimensional, cross-linked networks of water-soluble polymers that undergo physical transformation in the presence of water while maintaining structural integrity [62]. They are commonly classified as either physically cross-linked or chemically cross-linked gels [63]. Physically cross-linked hydrogels are reversible gels, less complex to produce as opposed to chemically cross-linked hydrogels, and do not involve the use of cross-linking agents during their production. The dissolution of reversible gels is inhibited by the presence of interactions that exist between polymer chains. Several methods exist that are used to bring forth physically cross-linked hydrogels viz., freeze-thawing, stereo-complex formation, ionic interaction, hydrogen bonding, and maturation [64]. Chemically cross-linked hydrogels are stable and do not dissolve in any solvent due to the presence of covalent bonds between different polymer chains [64]. They are relatively more complex to develop as opposed to physically cross-linked gels, as the existing variables such as gelation time, internal network pore size, and degradation time are not easy to decouple. Chemically cross-linked hydrogels exhibit great mechanical strength and can elicit a prolonged decomposition time. Several methods exist that are used to bring forth chemically cross-linked hydrogels, such as chemical cross-linking, grafting, radical polymerization, condensation reaction, enzymatic reaction, and high-energy radiation [63].

Hydrogels are capable of retaining a high amount of water or biological fluids therefore they can simulate natural living tissue, more effectively than any other synthetic biomaterials. Hydrogels can be considered as reversible/physical gels when in forming the linkages, forces such as ionic, hydrogen bonding, or hydrophobic forces play a role [65]. Polymerization and parallel cross-linking of multifunctional monomers are procedures that can be used to synthesize hydrogels. In addition, the formulation of polymer molecules that have reactive groups is a multi-step method that can be used [66]. Some of the merits of hydrogels include hydrophilicity, biocompatibility potential, controlled drug release, and smart drug delivery [67]. Hydrogels can be classified based on their physical state as solid, semi-solid, and liquids [45]. They are classified as either non-crystalline (amorphous), semi-crystalline, or crystalline [68]. Hydrogels may be categorized into four groups based on the presence or absence of electrical charge located on the cross-linked chains: anionic, cationic, amphoteric, and non-ionic. Based on durability, hydrogels can be classified as either biodegradable or non-biodegradable [43].

The setbacks that are associated with the drug delivery of hydrogels include the fact that hydrogels have a water-loving or hydrophilic polymeric core which is a challenge to delivering hydrophobic drugs. Furthermore, the weak tensile strength of hydrogels could cause the premature release of drugs [69].

Jalalvandi et al., reported on the fabrication of an injectable and biocompatible hydrogel system formulated for the delivery of a hydrophobic drug. The hydrogel contained a cyclodextrin moiety with a degradable polymer [70]. Low aqueous solubility of delivery of the insoluble drug for hepatocellular carcinoma A glycyrrhetinic acid (GA) molecule-modified curcumin-based hydrogel was developed to address the challenges [71].

Akiyoshi et al., reported a physically cross-linked glutaraldehyde for biomedical applications which was produced using the polymer pullulan bearing cholesterol. The ani-cancer drug, Adriamycin, was loaded by combining pullulan suspension with adriamycin [72]. A dual physically cross-linked hydrogel was prepared by simple two-step methods of copolymerization and freezing/thawing and consisted of poly(acrylamide-*co*-acrylic acid) (PAM-*co*-PAA) and poly(vinyl alcohol) (PVA) [73].

In a study, the preparation, characterization, and cytocompatibility of chitosan and poly(vinyl alcohol) (PVA) blends which were chemically cross-linked by glutaraldehyde for biomedical applications was reported [74]. In another study, a chemically cross-linked PVA–H_2_SO_4_ hydrogel film was synthesized by combining the covalent cross-linking of PVA chains and a film-casting process. It showed good elasticity, excellent mechanical strength, and ionic conductivity [75]. Contrary to other gels, in-situ, hydrogels are non-toxic, biocompatible, and degradable. Their mechanical properties and swelling behavior are some of their distinguishing features. Water-expanding materials called hydrogels have the ability to contain a lot of water inside their structure without dissolving. The tensile strength, % elongation to break, toughness, and Young modulus of hydrogels are among its mechanical characteristics [76].

The advantages, disadvantages, and applications of the discussed classes of cross-linked gels are summarized in Table 1.

## 4. Polymers and Cross-Linking Agents

Cross-linked gels contain cross-linked polymer chains whose properties govern the properties of the resulting gel [77]. Various polymers and crosslinkers have been utilized to formulate cross-linked gels of impeccable properties.

### 4.1. Natural Polymers

**Figure 3:**
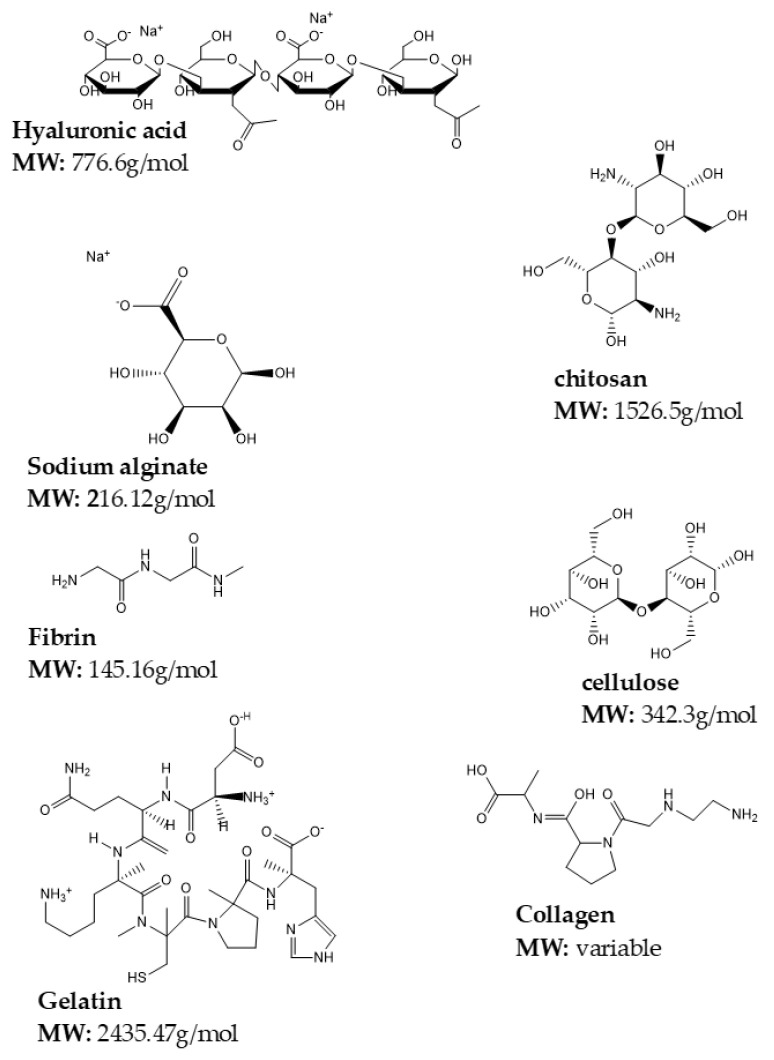
Chemical structures and molecular weights (MW) of naturally derived polymers used in the manufacture of cross-linked gels.

Natural polymers are materials that generally occur in nature, or are extracted from plants or animals [78]. Natural polymers include proteins such as collagen, gelatin, and fibrin and polysaccharides such as chitosan, alginate, cellulose, and starch +. These polymers are more biocompatible in comparison to synthetic polymers since their biodegradation is regulated by human enzymes such as lysozyme. Their advantage lies in that they are eco-friendly, have minimal and production costs [79]. Conversely, their limitations are that they have inadequate mechanical strength, immunogenicity, batch-to-batch variations, and transfer of pathogens from animal material [80]. These limits the use of natural polymers in medical applications and thus requires that they are combined with organic/inorganic molecules [81]. The chemical structures of some of the naturally occurring polymers utilized in gel formation are depicted in Figure 3.

#### 4.1.1. Chitosan

Chitosan is a highly applicable polysaccharide due to its biodegradability, biocompatibility, non-toxicity, antioxidant, anti-inflammatory, antifungal, and antibacterial properties [79,82]. The ability to respond to various environmental stimuli such as different pH and temperature conditions of physiological fluids has gained much attention to chitosan hydrogel worldwide [79]. Chitosan polymer is made of β (1–4)-linked 2-amino-2-deoxy-D-glucose (D-glucosamine) and 2-acetamido-2-deoxy-D-glucose (N-acetyl-D-glucosamine) units. Chitosan is insoluble at a pH greater than 6 and soluble at a pH below 6 due to the protonation of amine groups [83]. Chitosan is derived from partial or full deacetylation of *N*-acetylglucosamine copolymer from chitin residues, the degree of acetylation and the molecular weight of chitosan, determines its solubility, hydrophobic, and electrostatic properties [84]. The hydroxyl (-OH) and amine (-NH2) groups in chitosan’s chemical structure allow it to develop cross-linked hydrogen bonds with other polymers such as polyethylene glycol [84].

#### 4.1.2. Gelatin

Gelatin is a derivative of moderately hydrolysed indigenous collagens of animal skin, bones, and tendons. Gelatin is vastly used in biomedical applications [84]. It is a biocompatible, non-immunogenic, and non-toxic polymer, with high water solubility at body temperature and amphoteric nature [83]. These make it suitable for different applications such as in the manufacturing of contact lenses, improving mechanical properties of other polymers such as alginate, wound healing, tissue engineering, and drug delivery [83,85]. The structural alignment of gelatin-based materials is alike to the extracellular matrix and permits activation of platelet aggregation hence its suitability in hemostatic applications [86].

#### 4.1.3. Sodium Alginate

Sodium (Na) alginate is a hydrophilic, cationic polysaccharide consisting of (1–4)-linked β-d-man urinate (M) and its C-5 epimer α-l-guluronate (G) residues [87]. It is derived from extracts of varied bacteria and algae, it is well biodegradable, non-immunogenic, and non-toxic [84]. This extensively biocompatible polymer is used to make stable cross-linked gels for drug delivery systems. Bulut et al. developed polyvinyl alcohol grafted polyacrylamide/Na alginate/NaCMC microspheres by the emulsification cross-linking method that are used for the delivery of donepezil hydrochloride. [88,89]. The gel matrix formed in the presence of divalent cations, mostly Ca^2+^, making alginate widely usable in the encapsulation and controlled release of pharmaceutical materials, however, the porosity, permeability, and degradability of the gel matrix make it difficult to regulate the release of the encapsulated material [83,84]. Additional drawbacks include weak mechanical strength, and difficulties in sterilization, handling, and storage of solutions [81].

#### 4.1.4. Collagen

The biocompatible and biodegradable attributes of collagen polymer make it an excellent polymer for use in areas such as controlled drug delivery, inducing cell growth, preparation of plants, and pharmaceutical industries [67]. Collagen is one of the major structural elements of extracellular matrix (ECM) in bones, connective, and nerve tissues which are responsible for elastic strength, regulating cell adhesion, chemotaxis, migration, and tissue development. This polymer is the most abundant fibrous protein in the human body [90,91]. Collagen has challenges such as inconsistency, and ethical and cultural issues as they are derived from animal sources. To overcome these limitations, collagen type I scaffold was engineered in-vitro; this is biocompatible, biodegradable, extremely versatile, easy to use, and can make stable cross-linked networks [92].

#### 4.1.5. Cellulose

Cellulose is one of the most abundant natural polymers derived from wood, algae, and bacteria [93,94]. Cellulose is composed of a β -1,4-linked anhydro-D-glucose chain with abundant hydroxyl groups that offer versatile prospects. Cellulose does not dissolve in the mild aqueous solvent system and common organic solvents [93]. Characteristics of cellulose include high biocompatibility, low immunogenicity, in-situ flexibility and tensile strength, less porous structure, and toxicity. Additionally, it has high water retention >95%, ion exchange capacity, transparency, and permeability to liquids and gases making it appropriate for laxative effects, contact lenses, and wound drug delivery amongst others [95,96]. The design and synthesis of cellulose-based gels can be achieved by both chemical linking and/or physical cross-linking such as hydrogen bonding, hydrophobic interaction, and ionic complexations [93]. Zhang et al. designed a cross-linked gel polymer electrolyte by modifying cellulose with ally groups and reacted it with polyethylene glycol methyl ether methacrylate (PEGMA) to obtain PEG-grafted cellulose, pentaerythritol tetra(3-mercaptoproponate) (PETMP) that was used as a cross-linking agent to form the cross-linked network polyethylene-propylene copolymers which showed good mechanical properties, structure strength, and stability [94].

#### 4.1.6. Hyaluronic Acid

Hyaluronic acid (HA) is a water-soluble, bio-derived, and biodegradable polymer [81]. It is commonly excreted by fibroblasts and chondrocytes. Hyaluronic acid is an important part of the extracellular matrix and also functions in cell signaling and wound repair [97]. HA is inherently biocompatible and non-immunogenic and degrades in the presence of hyaluronidase, hindering its usefulness in certain applications [87]. HA can be modified with thiols, haloacetates, dihydrazides, aldehydes, or carbodiimide moieties to facilitate cross-linking into a gel [87]. HA-based hydrogels have been used in various biomedical applications including controlled drug delivery [98]. A recent study assessed the feasibility of HA hydrogels as carriers for controlled pulmonary drug delivery of inhalation powders and their finding showed that HA hydrogels could successfully be used as drug carriers for sustained pulmonary delivery [98].

#### 4.1.7. Fibrin

The formation of cross-linked gels from fibrin is based on its biological polymerization process with thrombin following vascular injury [99]. It forms cross-linked networks by enzymatic polymerization of its precursor, fibrinogen, via thrombin-catalyzed cleavage of fibrinopeptide A in the presence of factor XIII [87]. Fibrin-based cross-linked gels have been used in biomedical applications such as for wound healing [100], controlled drug delivery [101], and tissue engineering [102,103]. Murphy et al. engineered a fibrin-based hydrogel to simultaneously enhance the wound-healing properties and anti-inflammatory potential of entrapped human mesenchymal stem cell spheroids [100].

### 4.2. Synthetic Polymers

**Figure 4:**
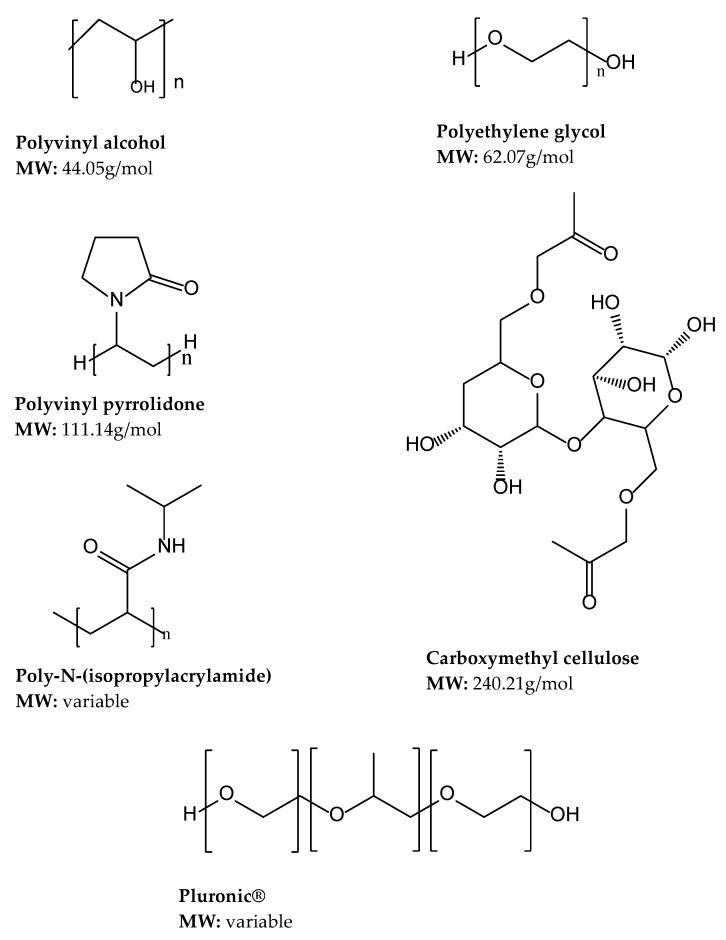
Chemical structures and molecular weights (MW) of various synthetic polymers used to manufacture cross-linked gels.

Synthetic polymers such as polyethylene glycol, polyacrylic acid, polyethylene oxide, and polyvinyl alcohol are used in their organic and inorganic forms [104,105]. Synthetic polymers are more hydrophobic and mechanically stronger than natural polymers, with an inactive cellular environment that forbids active cell binding resulting in low stem cell viability [106]. Additionally, synthetic polymers possess more reproducible physical and chemical properties compared to natural polymers [107]. However, due to their association with high immunogenicity [107], synthetic polymers are commonly used for short-term applications [108]. A summary of some of the synthetic polymers used in gel development are provided in Figure 4.

#### 4.2.1. Polyethylene Glycol (PEG)

Polyethylene glycol makes a good candidate for hydrogel formulation due to its high-water solubility and swelling index, biocompatibility, good gelation properties, and low immunogenicity [109,110]. These properties are essential in the central nervous system for use of nerve repair in spinal cord injury and inhibition of axon degeneration [104]. The limitations of PEG include poor cell affinity, reduced cellular response, and low cell adhesion [81]. PEG networks can be prepared by chemical cross-linking of reactions of difunctional PEGs and multifunctional cross-linking agents, radiation cross-linking of PEG chains to each other, which do not require toxic cross-linking agents but are difficult to control, and physical cross-linking of hydrophobic blocks of triblock copolymers carrying hydrophobic and PEG blocks [68].

#### 4.2.2. Polyvinyl Alcohol

Polyvinyl alcohol (PVA) is a widely used polymer with good thermal stability, biocompatibility, biodegradability, mechanical strength, viscoelasticity, and hydrophilicity [67,111]. It is non-toxic, pH stable, and has coagulating ability; it is thus suitable for cell growth but limited by its inability to control fast-flowing haemorrhages [86]. High molecular weight PVA forms strong, elongated, rigid, and highly crystalline film structures. However, PVA does not support cell proliferation and attachment, hydrophilicity and elasticity are limited, and, thus for wound healing, it requires a combination with an antimicrobial agent to achieve antibacterial activity [81,112]. PVA shows viability as a biological drug delivery matrix and can be cross-linked using various methods to improve its properties. The methods include cross-linking of linear PVA chains with glyoxal, glutaraldehyde, or borate and preparation of semicrystalline gels by freezing and thawing of aqueous PVA solutions that allows for network structures cross-linked with pseudo-permanent crystallites. The freezing method is regarded as pure as it does not require a crosslinker and thus is most preferred [68].

#### 4.2.3. Polyvinyl Pyrrolidone (PVP)

Polyvinyl pyrrolidone is one of the most popular water-soluble, biodegradable, biocompatible, and extremely low cytotoxic synthetic polymers [113]. It has been mainly used to enhance the solubility of drugs and has been reported to have enhanced the solubility of curcumin by 880-fold in solid dispersion prepared by the coevaporation of curcumin and PVP ethanol [114,115]. Although its biodegradability is a desired attribute, PVP rapidly dissolves in water, significantly hindering its use in sustained release formulations [114]. To circumvent this drawback Contardi and colleagues’ fabricated fibrous PVP-based hydrogels as skin dressing loaded with p-coumaric and ferulic acid in skin damages and disorders [114]. Other studies found that blending PVP and carboxymethyl cellulose (CMC) yielded a hydrogel with enhanced mechanical properties and enhanced biodegradability [116].

#### 4.2.4. Carboxymethyl Cellulose (CMC)

CMC is a cellulose derivative derived from the partial substitution of the cellulose hydroxyl group by the carboxymethyl group [117]. CMC is used in cross-linked gels in fields such as wound healing, drug delivery, plant breeding, and tissue engineering attributed of its biocompatibility, biodegradability, water solubility, abundance, low cost, and environmental friendliness [67,74]. The insufficient amount of hydroxyl group in the CMC chain results in difficulty in gelation of cellulose solutions and reduced mechanical strength, thus chemical cross-linking agents are necessary to improve these properties [118]. CMC has been cross-linked with metal ions whereby Liu et al. manufactured a self-recoverable, anti-fatigued, and self-healing poly (N-acryloyl glycinamide) (PNAGA)/CMC hydrogel using Fe^3+^ ions cross-linked CMC and hydrogen bonds cross-linked PNAGA as the first and second network, respectively [117].

#### 4.2.5. Poly (N-isopropyl acrylamide)

Poly (N-isopropyl acrylamide) (pNIPAAM) is a thermosensitive polymer that undergoes polymer phase separation at increased temperature and reaches a lower critical solution temperature (LCST) at about 33 °C [119]. pNIPAAM has been useful for pulsatile drug release in response to temperature for drug delivery systems [120]. At 32 °C linear pNIPAAM produces unstable hydrogels that substantially collapse with increasing temperature above the LCST. This thermo-responsive nature is due to the strong hydrogen bonds between the polymer and water molecules and the specific structural molecular alignments of these bonds [121,122]. Specific molecular alignment is required for the hydrogen bonds between the water and amide group due to the hydrophobic *N*-isopropyl residues in pNIPAAM [122].

#### 4.2.6. Pluronics^®^

Pluronics^®^, also called poloxamers, are a group of synthetic triblock copolymers composed of hydrophobic polypropylene oxide (PPO) and hydrophilic polyethylene oxide (PEO) set in a manner of PEO-PPO-PEO. Pluronics^®^ have outstanding biocompatibility and amphiphilic properties widely used in drug delivery, disease diagnosis, and treatment [123,124]. Hydrophilic Pluronic^®^ can be used as stabilizers and retention agents due to their good biocompatibility while hydrophobic Pluronic^®^ are compatible and essential for drug loading [123]. Pluronics^®^ are thermosensitive polymers that exist in aqueous solutions at LCST without any phase separation, and undergo sol-gel transition at increased temperature once in contact with the body [125,126]. The challenge with Pluronic^®^ lies in the weak mechanical strength at low concentrations, lack of mucoadhesion, and lower gelation temperature at high concentrations [125,127]. A study by Boonlai et al., has developed a promising injectable in-situ gelling matrix for periodontal therapy drug delivery by mixing Pluronics^®^ 407 with polyacrylic acid held by hydrogen bonds, which was able to achieve desired mucoadhesive properties and gelation at body temperature [127].

### 4.3. Crosslinkers

Crosslinkers are molecules with at least two reactive functional groups (e.g., primary amines, sulfhydryls, carbonyls, carbohydrates, and carboxylic acids) that allow for the formation of bridges between polymeric chains [128]. The crosslinker agent controls the structure of the polymer matrix. It leads to the aggregation and connection of functional monomers to each other. Crosslinkers cause polymerization and molecular template separation, without displacement of functional monomers [129]. Selecting the appropriate cross-linking agent enables the polymerization to be carried out as desired and may result in cross-linked gels of desired attributes.

#### 4.3.1. Natural Crosslinkers

**Figure 5:**
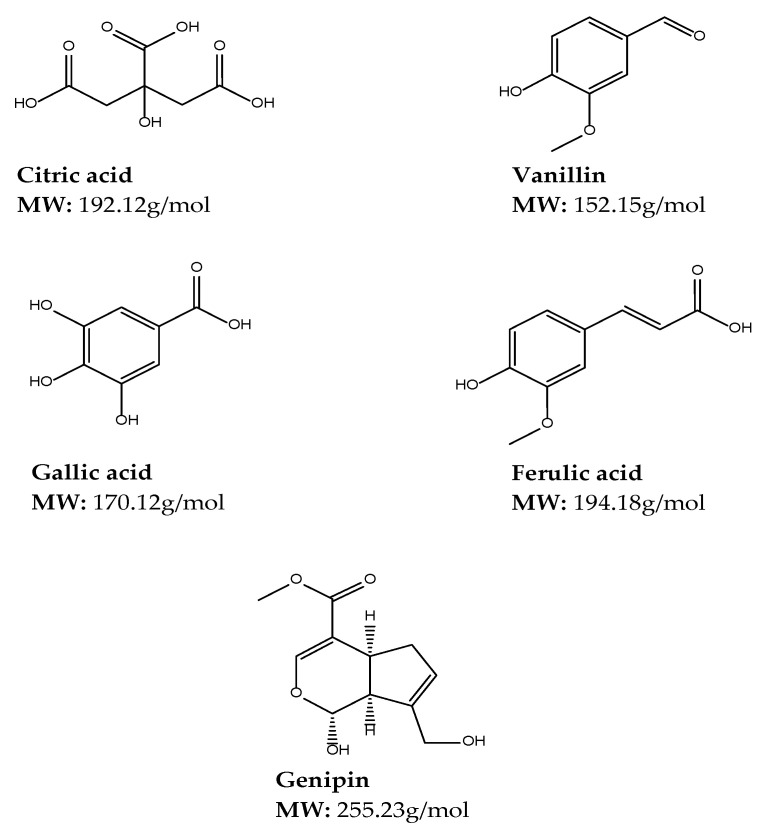
Chemical structures and molecular weights (MW) of various natural crosslinkers used to manufacture cross-linked gels.

Genipin, enzymes, and citric acid are some of the frequently used natural crosslinkers due to their low toxicity [130]. These cross-linking agents have improved biodegradability, biocompatibility, and water solubility compared to synthetic crosslinkers and have recently gained much attention to form biocompatible materials, inclusive of other crosslinkers such as gallic acid, ferulic acid, vanillin, flavonoids (catechins, pro-anthocyanidins) and desmosine analogs [131]. The chemical structures and molecular weights of some of the naturally occurring crosslinkers utilized in cross-linked gel preparation are depicted in Figure 5.

##### Citric Acid

Citric acid (CA) is a non-toxic metabolic product of the body from the Krebs cycle that has been approved by the food and drug administration for use in food products [132]. CA has been used as a cross-linking agent for cross-linking polymers such as starch and xanthan gum. CA can also be used as a hydrolytic and plasticizer [133]. CA with a hydroxyl group and 3 carboxyl group is a safe, non-toxic, and biologically friendly crosslinker used for cross-linked gels such as aerogels [134]. The multi-carboxylic structure of CA allows for interactions with hydroxyl groups from other polymers which reduce water resistance due to unavailable OH groups. From this interaction, strong hydrogen bonds could also be formed and prevent recrystallization and retrogradation, hence their wide use with starch [132]. Kuenzel et al. reported that CA carboxylic groups can also interact with amines groups in a temperature-induced condensation to form a chitosan/CA binder for LiNi_0.5_Mn_1.5_O_4_ (LNMO)-based positive electrodes [135].

##### Vanillin

Vanillin is the main product of vanilla bean extract. The structure of vanillin has an aldehyde group that forms a Schiff base with primary amines which are responsible for hydrogen bonds with hydroxyl or amine groups. Thus, this offers the formation of reversible cross-linked networks and self-healing hydrogels with low-temperature stability that is altered with increased temperature due to breaking hydrogen bonds [130]. Vanillin is a popular flavouring agent widely used in areas such as food, beverages, perfumery, and pharmaceutical industries. Vanillin also has antioxidant properties that are used to improve the antioxidant properties of polymers and have less harmful health effects compared to synthetic antioxidants [136]. A study by Brito et al. demonstrated that vanillin structured stronger and more mechanically stable chitosan-based oleo-gels at low concentration, thus vanillin appears to be a promising candidate as saturated or trans-fat replacers in processed foods and was effectively used in a proof-of-application set of experiments with cookies [137].

##### Gallic Acid

Gallic acid (GA) also known as 3,4,5-trihydroxybenzoic acid is tannin and is the major phenolic compound in plants, fruits such as grapes, berries, mango, areca nut, walnut, and green tea. GA has advanced anti-carcinogenic, anti-viral, antibacterial, antifungal, anti-inflammatory, and anti-malarial properties [138,139]. Additionally, GA possesses antioxidant activities equal to a well-established antioxidant vitamin C [140]. Xie et al. suggest that it is a valuable approach to graft GA onto chitosan to reduce the intra- and intermolecular hydrogen bond networks and increase the antioxidant ability of CS [138]. Chemical or enzyme-modified GA-chitosan conjugate presented improved bioactivities such as antioxidant, antibacterial, anticancer, and inhibitory effects on digestive enzymes when compared to free GA or biopolymer [139].

##### Ferulic Acid

Ferulic acid (4-hydroxy-3-methoxycinnamic acid) is a secondary metabolite of the biosynthesis of lignin, originating from phenylalanine and tyrosine through the Shikimate pathway. Ferulic acid (FA) can be found in cereal, fruits, vegetables, and plant tissues in its natural free from either monomers, dimers, or polymers as well as esters through condensation with hydroxyl acids, alcohols or saccharides or as amides condensed with amines [141]. The cross-linking of FA with polysaccharides and proteins during cell wall synthesis takes place when FA deposits are incorporated utilizing an ester bond to primary alcohol or arabinose side chains in the arabinoxylan polysaccharides cell wall [142]. FA has been merged into a biodegradable polymer as a pendant group to enhance antioxidant properties, specifically for tissue engineering applications [141]. FA is a phenolic compound that has anti-inflammatory, antimicrobial, and anticancer properties; it also acts as a photo-protective and antioxidant agent in biomedical and cosmetic applications. It is an ultraviolet absorber and free radical scavenger that can prevent harmful radiation effects. However, its limitations are that it endures thermal, air, and light-induced decomposition through the anticipated decarboxylation mechanism, thus reducing its efficiency [143].

##### Genipin

Genipin has replaced glutaraldehyde and other carbodiimide crosslinkers due to the expanded biochemical significance of genipin-cross-linked gels [144]. It is produced from the hydrolysis of geniposide extracted from the fruits of Gardenia jasminoides Ellis [145]. Genipin is a natural and biodegradable alternative to synthetic cross-linking agents. Its non-toxicity and good biocompatibility allow for numerous applications in the biomedical and food fields. It is more eco-friendly and less biohazardous than glutaraldehyde [145]. It forms stable crosslinks following a spontaneous reaction with primary amino groups of polymers [145]. An example of such cross-linking is seen when genipin is allowed to react with primary amine groups of chitosan, resulting in crosslinks between chitosan molecules.

#### 4.3.2. Synthetic Crosslinkers

**Figure 6:**
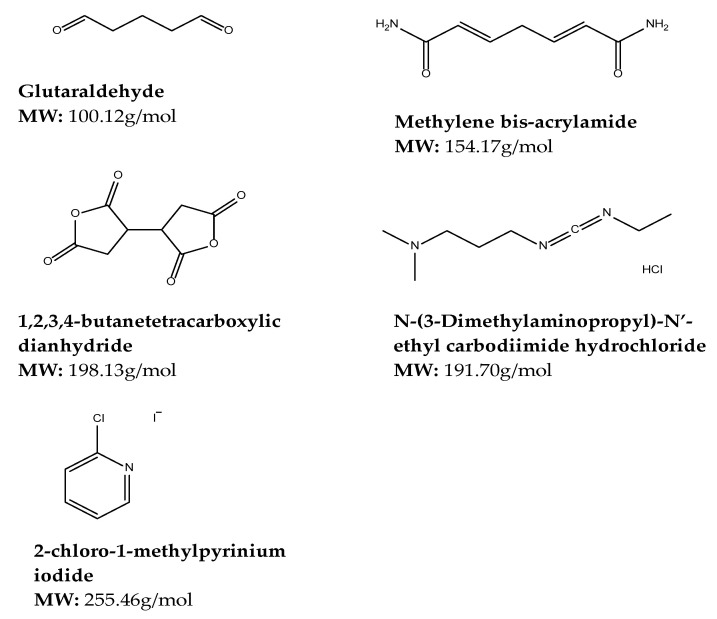
Chemical structures and molecular weights (MW) of various synthetic crosslinkers used to manufacture cross-linked gels.

Synthetic crosslinkers are widely acceptable for applications such as tissue engineering and regenerative medicine due to their fast effect on modifying the polymeric backbone and high degree of cross-linking. Some of the crosslinkers include glutaraldehyde (GA), 1,4-butanediol di glycidyl ether (BDDGE), 1-ethyl-3-[3-dimethylaminopropyl] carbodiimide hydrochloride (EDC) [146]. Other synthetic cross-linking agents include di-hydrazides, epoxy-based compounds, carbodiimide, boric acid, sodium TriMet phosphate, N, N′-methylene bis-acrylamide, and poly-carboxylic acids. These crosslinkers present moderate or low toxicity except for glutaraldehyde [130]. Common limitations of these crosslinkers include toxicity, low biodegradability, biocompatibility, and aqueous solubility as compared to natural crosslinkers [131]. The chemical structures and molecular weights of some of the synthetic crosslinkers utilized in cross-linked gel preparation are depicted in Figure 6.

##### Glutaraldehyde

Glutaraldehyde (GA) is a crosslinker for biomedical applications such as enzymes and hydrogel synthesis [147]. It is a dialdehyde whose aldehydic groups are highly reactive and can form covalent bonds with amine, thiol, phenol, hydroxyl, and imidazole functional groups [147]. It reacts with the primary amine groups of CS to form Schiff bases [148]. The cross-linking of hydroxyl groups with GA is often carried out in an acidic environment as the presence of acid catalyzes the reaction between the hydroxyl and aldehyde functionalities. On the contrary, the cross-linking reaction between amines and glutaraldehyde is slower in acidic conditions compared to neutral and basic conditions [147]. For many years, glutaraldehyde was the most used cross-linking agent due to its ease of access and cost-effectiveness [145]. However, the main drawback of using glutaraldehyde is its toxicity. Cytotoxicity studies in rats and mice from as early as 1993 found that glutaraldehyde resulted in a spectrum of necrotic, inflammatory, and regenerative lesions confined to the upper respiratory tract following exposure to glutaraldehyde by inhalation for up to 13 weeks [149]. Therefore, its use has been replaced by other crosslinkers as it is desirable to use a nontoxic cross-linking agent.

##### Methylene-bis-acrylamide

The *N*,*N*′-methylene-bis-acrylamide (MBA) is a common crosslinker in water-soluble polymers, which can integrate amide groups in hydrogels [150], and has two terminal vinyl groups that can react with radicals [151]. MBA is a bifunctional molecule popular for producing hydrogels based on acrylic acid (AAc) and acrylamide (ACL), however, the synthesis of hybrid materials based on polysaccharides, proteins, and monomers such as soy protein/Aac, alginate,/chitosan/ACL and gel/dextrin/ACL through cross-linking with MBA has also occurred [130]. Although commonly used for the formation of methacrylate networks, MBA’s use as crosslinkers is limited due to their poorly aqueous-soluble nature [131]. Mohana et al. synthesized a novel superabsorbent in an aqueous solution by copolymerization of the respective monomers using MBA as a crosslinker and ammonium persulfate (APS) as the initiator [152]. While Alavarse et al. states that good compatibility and non-toxic effects on some cells were observed in hybrid hydrogels produced with MBA cross-linking. Additionally, these MBA-based hydrogels demonstrated suitability for biomedical applications such as tissue engineering and the release of co-enzyme A, verapamil, amoxicillin, quercetin, and cephalexin [130].

##### Polymerizable Polyphosphate

Polymerizable polyphosphate (PIOP) crosslinker is produced by ring-opening polymerization of 2-i-propyl-2-oxo-1,3,2-dioxaphospholane with 2-(2-oxo-1,3,2-dioxaphosphoroyl-oxyethylmethacrylate). PIOP shows degradation that has a direct proportionality to the pH of the media in an aqueous medium, whereby a complete degradation was observed at pH 11 within 6 days. Iwasaki et al. prepared a non-cytotoxic hydrogel by radical polymerization of poly (2-methacryloyloxyethyl phosphorylcholine) using PIOP as a crosslinker when evaluated with v79 cells [131].

##### 1,2,3,4-butanetetracarboxylic Dianhydride (BTCA)

1,2,3,4-butanetetracarboxylicdianhydride (BTCA) is a synthetic crosslinker with two acid anhydride groups that are easily cross-linked with functional groups such as isocyanate and hydroxyl [153]. BCTA appears to have potential in replacing traditional aldehydes. BTCA can crosslink with functional groups such as amine and hydroxyl to form amide or ester linkages that reduce water resistance [154]. The limitations of BTCA use in the textile industry include high cost, hazardous effect of phosphorus-containing catalyst, and loss of mechanical strength in the presence of poly-carboxylic acid, hence the requirement of a co-catalyst in the finishing system [155]. Kono et al. states that biodegradable hydrogels were prepared by homogenous solution phase cross-linking of polysaccharides such as cellulose and chitosan, cotton, and paper using BTCA as a cross-linking agent and 4-dimethylaminopyridine as a reaction catalyst where BTCA showed to be an exceptional crosslinker for polysaccharides that requires no derivatization procedures under mild conditions [153], thus improving the mechanical and barrier properties of polysaccharides [154].

##### *N*-(3-Dimethylaminopropyl)-*N*′-ethyl Carbodiimide Hydrochloride

*N*-(3-Dimethylaminopropyl)-*N*′-ethyl carbodiimide hydrochloride (EDC) is a competent cross-linking agent which has been utilized for cross-linking aqueous-soluble polymers through the formation of amide bonds [131]. EDC is zero-length which means the agent itself is not fused in the macromolecule. An amine-reactive O-acylsourea intermediate is established by joining the amine to the carboxylic acid group of glutamic and aspartic amino acid of the protein molecule using protonated carbodiimide. Additionally, the O-acylisourea intermediate can be used to enhance the cross-linking competence of EDC by reacting with *N*-hydroxy-sulfo-succinimide (NHS) to develop NHS-reactive NHS-ester coupled with the release of 1-ethyl-3(3-aminopropyl) urea which can be eliminated by rinsing [156]. Although non-toxic, highly compatible, and water-soluble, 2-chloro-1-methylpyrinium iodide is preferred over EDC in hyaluronic acid films since the use of EDC introduced *N*-acylurea groups [131].

##### 2-chloro-1-methylpyrinium Iodide

Additionally, 2-chloro-1-methylpyrinium iodide (CMPI) also known as Mukaiyama reagent is a room-temperature stable connector reagent used in the synthesis of peptides of biological products. CMPI’s benefits include low toxicity, water solubility, simple reaction conditions, short reaction time, high yields, and cost-effectiveness [157]. Zafar et al. states that CMPI facilitates the intra and intermolecular ester bond arrangement among carboxyl and hydroxyl groups of hyaluronic acid chains, whereby improved hydrolytic degradation resistance and higher cross-linked density are revealed by CPMI cross-linked hyaluronic acid films in comparison with 1-ethyl-(3,3 dimethylaminopropyl) carbodiimide hydrochloride (EDC) cross-linked films [131]. Yeh et al. discovered that CMPI is an efficient zero-length cross-linker of gelatin that can activate carboxylic acid sodium salt under heterogeneous reaction [157].

The advantages, disadvantages and effects of the natural and synthetic polymers discussed herein are summarized in Table 2 and Table 3, respectively, together with cross-linking agents or factors used for gelation.

## 5. Mechanisms of Gelation

**Figure 7:**
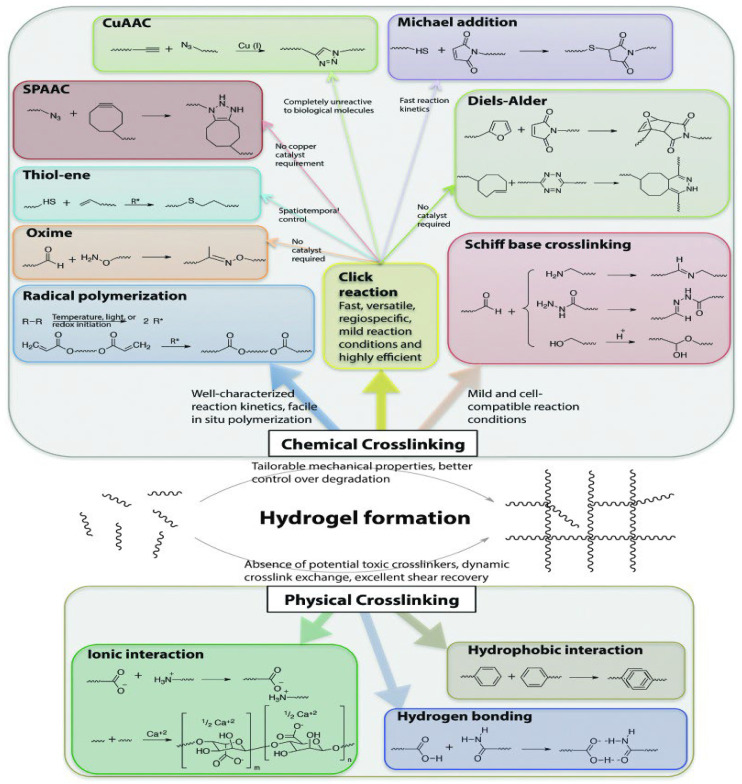
The various mechanisms of cross-linking that result in gelation [Obtained and reproduced without any changes from [87] and The Royal Society of Chemistry in accordance with Creative Commons Attribution License (CC BY)].

There are various mechanisms of gel formation from the initial solution state depending on the type of crosslinking, as depicted in Figure 7. Chemical cross-linking is an irreversible process that produces stable and strong gels [173], whereas physical cross-linking produces gels that are less stable, with relatively poor mechanical properties, and undergo a reversible swelling process in response to the environmental conditions [87].

Physically cross-linked gels are created by changes in environmental conditions whereby gelation often occurs under the influence of pH, temperature and other stimuli that could influence interactions between polymers [174]. Ionic interactions, hydrogen bonding, crystallization, and hydrophobic association are physical techniques utilized to crosslink polymers into reversible gels [175]. Ionic cross-linking usually occurs between two molecules or polymers of opposite charges [176]. On exposure to counter ions [177], a gel with potential for biodegradability is formed [87]. Hydrophobicity-driven gelation occurs in thermo-responsive hydrogels. Polymers with both hydrophilic and hydrophobic domains, referred to as amphiphiles, exhibit hydrophobic interactions [128]. Cross-linked gels can also be prepared by the formation of crystallites, which act as physical crosslinks for network formation [87]. In this mechanism, an aqueous polymer solution is frozen and then thawed at room temperature. This process is often referred to as freezing-thawing cycling and forms crystallites and hydrogen bonding between polymer chains, resulting in a gel with enhanced mechanical properties such as gel strength [178].

Chemically cross-linked gels comprise covalent bonds among polymer chains are formed via radical polymerization, Click reactions, Schiff base cross-linking reactions and enzymatic cross-linking of complementary groups [175]. Free radical polymerization is the most commonly used mechanism of hydrogelation [179]. The mechanism is mainly utilized to synthesize polymers from monomers containing carbon double bonds. The method consists of three main steps; (1) initiation, (2) propagation, and (3) termination to end the polymer chain propagation [180]. Click reactions are a class of chemical reactions that are fast, versatile, regiospecific, and highly efficient [87,181]. Click reactions are attractive tools for synthesizing cell-compatible gels due to the high regiospecificity and chemo-selectivity, mild reaction conditions, and simple modification of structural and mechanical properties using stoichiometry, attributed to their versatility [87]. The various click reactions that have been used to crosslink polymers include (1) copper-catalyzed azide-alkyne cycloaddition (Cu-AAC) reactions; (2) copper-free click reactions (Diels–Alder (DA) reactions, Strain-promoted azide-alkyne cycloaddition (SPAAC) reactions, radical mediated thiol-ene reactions, and oxime-forming reactions) [181]. Schiff base cross-linking reactions are reactions between molecules containing amine, alcohol, or hydrazide moieties and an aldehyde molecule [87]. The reactions involve a dynamic covalent amine bond formation via the cross-linking of amine groups and aldehyde groups and depend on the pH, temperature, and type of aldehydes/amines involved in the reaction [182,183]. Enzymes form covalent bonds between polymers, they are significantly sensitive and specific in chemical reactions [80]. The majority of enzymes involved in polymer cross-linking are common enzymes that catalyze reactions in the body [175]. Plasma Amine Oxidase and Lysyl Oxidase are examples of human enzymes that have been used as crosslinkers to develop biocompatible, in-situ gelling hydrogels [175].

## 6. Opportunities for Cross-Linked Gels in CNS Drug Delivery

The review highlights the opportunities in non-conventional approaches as the standard routes are currently heavily explored and reported for challenges faced with delivering the drug across BBB.

### 6.1. Non-Standard Routes of Administration for CNS Drug Delivery

Drug delivery strategies to the CNS can be implemented by several administration routes including systemic delivery, invasive local delivery (intrathecal and intraparenchymal delivery) as well as non-invasive administration routes (intranasal, oral, and peripheral delivery). Fortunately, cross-linked gels possess properties and characteristics that make them attractive candidates for application in direct drug delivery to the brain. They have been subjects of scrutiny in specialized drug delivery techniques such as intraparenchymal, intrathecal, and intranasal drug delivery.

#### 6.1.1. Intraparenchymal Drug Delivery

Intraparenchymal drug delivery is also referred to as intracranial or intracerebral injection. It is achievable by using natural or synthetic polymer-based systems, which provide controlled, timed and long-lasting drug delivery as the system degrades [33]. Cross-linked polymer systems, especially hydrogels, have been studied extensively for intraparenchymal delivery of drugs. Both natural and synthetic polymers have been used in the form of wafers, injectable hydrogels, and implantable hydrogel scaffolds to encapsulate drugs [33]. Sellers et al. demonstrated the application of cross-linked gels as candidates for intraparenchymal drug delivery by formulating an injectable F-127 hydrogel depot containing poly(lactic-co-glycolic acid) microspheres loaded with hirudin, a thrombin inhibitor, for traumatic brain injuries [184]. The hydrogel system resulted in a prolonged drug release of the drug, improving overall CNS recovery [184]. Similarly, hydrogel scaffolds have also been studied for controlled CNS drug delivery. Hydrogel scaffolds are designed to mimic target tissue environments. Nguyen et al. formulated an implantable hydrogel-based scaffold comprised of collagen hydrogel loaded with aligned poly (ε-caprolactone-*co*-ethyl ethylene phosphate) electrospun nanofibers in a 3-D configuration [185]. The nanofibers-hydrogel scaffolds showed great biocompatibility, it supported functional neuronal reconnections, remyelination, and functional recovery following spinal cord injury [185]. Although intraparenchymal implants may be challenging to implement in humans in clinical scenarios, they remain a field for exploration.

#### 6.1.2. Intrathecal Drug Delivery

In intrathecal drug delivery, drugs can also be administered directly to the CSF by intrathecal injection, potentially evading the shortfalls of systemic drug delivery to treat CNS diseases [33]. Although substances injected directly into the CSF bypass the BBB, they are still subjected to ependymal cells of the choroid plexus forming the BCSFB [33]. This limits the tissue penetration despite their widespread distribution in the CSF [186,187]. One of the earliest reports of successful intrathecal drug delivery was by LeBel et al. in 1999, when they injected leptin directly into the CSF in the lumbar regions of animal models [188]. Although this drug delivery technique is extremely invasive, it is a promising candidate for direct-to-brain delivery of drugs in CNS emergencies. Cross-linked polymer systems can be utilized and manipulated to cause in-situ gelation triggered by the CSF microenvironment to deliver drugs in a sustained, controlled manner.

#### 6.1.3. Nose-to-Brain Drug Delivery

Nasal drug delivery is a non-intrusive technique of delivering drugs to the respiratory system, the central nervous system (CNS), and/or systemic circulation [189]. Intranasal delivery offers direct to brain drug delivery via intracellular and extracellular pathways, involving transcellular, paracellular, and extracellular transport mechanisms alongside the olfactory and trigeminal nerve fibers [190]. It has many advantages over other systemic delivery routes, such as its non-invasive attribute, fast onset of action, and in most cases, reduced side effects due to reduced systemic exposure [190]. Despite the potential enhancement of clinical efficacy with intranasal administration, drug delivery is often limited by the nasal properties of the nasal mucosa such as mucociliary clearance and surface charge. Mucociliary clearance of drugs is an important challenge when drugs are delivered intranasally [189,191]. Nasal absorption is critical for intranasally administered drugs to exhibit their action [191]. A drug’s lipophilicity, molecular weight, and charge are critical in their absorption intranasally. Nanotechnology has been explored to increase the delivery of drugs to the CNS [190].

Drugs that cannot cross the naso-mucosal membrane are subject to mucociliary clearance [189,191]. This drawback can be overcome by the development of a mucoadhesive system [191]. As such, polymers such as chitosan and alginate have been impregnated with drugs to produce sustained drug delivery while also preventing efflux of said drugs by BBB efflux transporters such as Pgp as well as preventing enzymatic degradation of the active pharmaceutical ingredients outside the CNS [190]. Melamane and colleagues developed a thermosetting hydrogel loaded with lamotrigine for intranasal administration to manage and treat generalized epilepsy [192]. The hydrogel was prepared by physically cross-linking Pluronics^®^ 407 (L127), Pluronics^®^ 188 (L68), and Carbopol^®^ 974P NF(C974) [192]. Liu et al. took it a step further by using carboxymethyl chitosan nanoparticles as carriers to successfully deliver carbamazepine intranasally to bypass the blood-brain barrier [191]. Their use of carboxymethyl chitosan, whose parent compound is a polymer that can be cross-linked with other polymers to form gels, indicates that cross-linked gels may provide a plethora of drug delivery platforms with different polymer compositions and particulate sizes.

#### 6.1.4. Eye-to-Brain Drug Delivery

The retina of the eye shares the same embryological origin and vasculature as the CNS [193]. The inner blood-retinal barrier (BRB) and BBB are quite similar; so are the aqueous humor of the eye and the brain’s CSF [193]. Hence, drug delivery to the CNS via ocular administration is considered crucial. However, the conventional ophthalmic formulations exhibit short pre-corneal residence time and poor bioavailability due to non-productive absorption, impermeability of drugs through the cornea, drainage, induced lachrymation, and tear turnover [194]. To circumvent these challenges, cross-linked gels have been studied in an attempt to develop stable formulations capable of delivering drugs in a sustained manner [194].

Solution-based ophthalmic formulations such as eyedrops demonstrate low bioavailability because of rapid lacrimal drainage in the eye. Common interventions involve frequent instillation of concentrated solutions to reach the desired therapeutic drug level within the eye. However, the drainage of drugs through the nasolacrimal duct may result in systemic absorption causing some undesirable side effects. In-situ gelling solutions have been prepared to overcome this challenge. Through careful selection of polymers, smart drug delivery systems have been shown to improve bioavailability in ocular drug delivery. For instance, Gupta et al. prepared an in-situ gelling solution with a combination of carbopol and chitosan polymers. Both polymers exhibit sol-to-gel transitioning in response to increasing pH. In-vivo findings of the in-situ gelling system displayed a slow onset of action followed by an intense biological response over a prolonged period, i.e., controlled ocular delivery [195]. A novel flunarizine hydrochloride-loaded organogel for intraocular drug delivery in-situ was developed by Dai and colleagues [196]. The organogel was prepared using soybean oil, stearic acid, and N-methyl-2-pyrrolidinone (NMP) and resulted in prolonged drug residence time in the plasma and brain tissues of the in-vivo models following intraocular delivery [196].

### 6.2. Nanotechnological Interventions

#### 6.2.1. Nanocomposite Cross-Linked Gels

**Figure 8:**
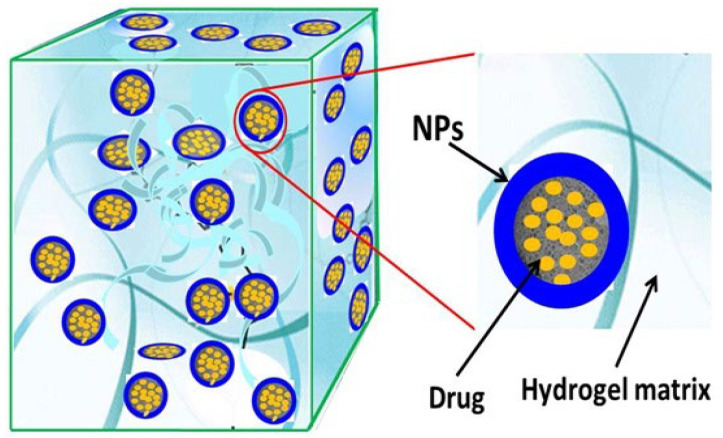
Schematic of the composition of nanocomposite cross-linked gels with a hydrogel dispersion matrix and drug-loaded nanoparticles. [Obtained and re-produced without any changes from [172] and Nanomaterials MDPI in accordance with Creative Commons Attribution License (CC BY 4.0)].

These are simply cross-linked 3-D networks formed in the presence of nanostructures by either physical or chemical cross-linking [197]. They can be formed by the addition of nanomaterials to a cross-linked gel matrix, which either absorbs the nanoparticles into the matrix or disperses them homogeneously inside the matrix and also by entrapping nanoparticles inside the gel matrix as depicted in Figure 8. These nanomaterials act as nanofillers within the matrix and enhance the intrinsic and extrinsic properties of the gel matrix such as mechanical and swelling/deswelling properties [197]. Many known carbon-based nanomaterials, polymeric nanoparticles, and metallic nanoparticles have been incorporated as nanofillers in hydrogel matrices to obtain nanocomposite gels [198]. The addition of nanofiller to the gel matrix contributes to the unique properties of cross-linked gels and broadens their applications in DDS as they allow for nanocomposite gels design in various forms, such as membranes, and sheets, hollow tubes, and bellows [197].

Nanofillers such as nanoclays [199], polymeric nanoparticles [200], and nano-silicates [201] have been investigated in various studies for their incorporation into nanocomposite cross-linked gels for drug delivery and other biomedical applications. Nanofillers have been widely used to improve drug dissolution rate, and drug stability while simultaneously modifying the DDS [197]. They have also been associated with high retention capacities, and colloidal, and swelling properties, showing their applicability in drug delivery [197]. Prolonged and slow drug release has also been reported in-vitro and in-vivo translating into improved bioavailability [202,203]. For example, Tao et al. prepared a nanocomposite hydrogel loaded with paclitaxel (PTX) nanoparticles to inhibit the recurrence of glioma. The hydrogel demonstrated sustained release of PTX and had a uniform distribution of the nanoparticles [204]. The slow and efficient drug release indicated an improvement in drug bioavailability in-vitro [204].

#### 6.2.2. Nano-Sized Cross-Linked Gels

Nanogels are essentially nanosized hydrogels, typically spherical within the 20–200 nm size range [205,206,207,208]. They possess characteristics of hydrogels, including the ability to swell, holding considerably large amounts of water without dissolving in the aqueous media, and are composed of cross-linked polymers. Additional characteristics including size, charge, porosity, hydrophilicity, softness, and degradability are modifiable by varying the chemical composition of nanogels to attain desired characteristics [10,11].

**Figure 9:**
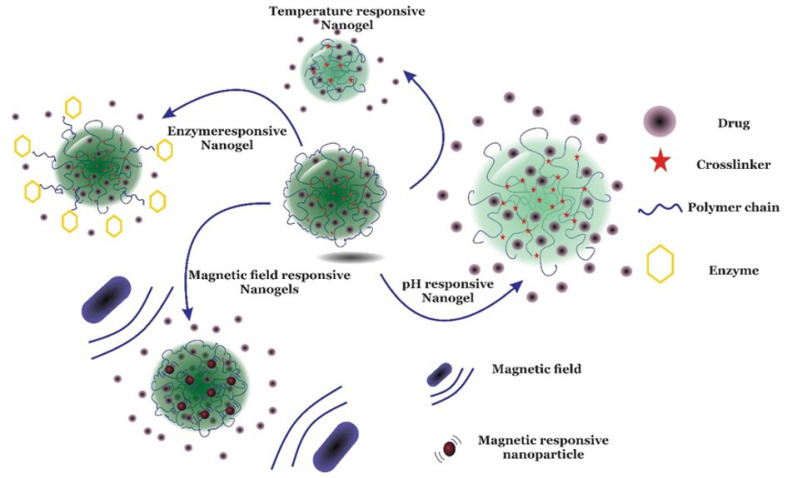
Schematic of drug release from nanogels in response to various stimuli [Obtained and reproduced without any changes from [183] and Gels MDPI in accordance with Creative Commons Attribution License (CC BY 4.0)].

Nanogels offer a better advantage in target specificity and reachability over macro-sized cross-linked gels due to their size and soft nature [11]. Their nano-scaled size enables them to readily reach alternative tissues compared to larger hydrogels [209], while their softness enables them to behave differently from solid and self-assembled drug delivery systems such as polymer-based nanoparticles, micelles, and liposomes [11]. Nanogels can be classified based on their responsive behavior, which can be either stimuli-responsive or non-responsive. Non-responsive nanogels swell when in contact with water, while stimuli-responsive nanogels are activated by contact with environmental conditions, such as temperature, pH, magnetic field, and ionic strength, which act as stimuli [206]. Any changes in any of these environmental factors, which act as stimuli, will lead to an alteration in the behavior of the nanogels as a response to causing drug release, as depicted in Figure 9 [206].

Nanogels which are responsive to a combination of various stimuli are termed multi-responsive nanogels, which can be designed with multi-drug release systems to control the release behavior of each drug separately [210]. The group of researchers designed a polymer hydrogel that specifically responded to electromagnetic radiation. The results of intravital imaging of rats showed that fluorescently labeled lamotrigine nanogels are enriched in the rat brain 3 h after intravenous injection, indicating that lamotrigine nanogels are characterized by enhanced BBB penetration.

Polarized monolayers of bovine brain microvessel endothelial cells for use in the delivery of oligonucleotides (ODN) to the brain using nano-PEG-cross-PEI nanogels were evaluated by Vinogradov and co-workers [211]. This was the first-ever reported nanogel. According to the results, ODN encapsulated in nanogel was successfully transmitted from the apical to the basolateral side of the BBMEC monolayers. Furthermore, the study indicates nanogel’s ability to shield ODN from enzymatic cellular breakdown. Last but not least, the in-vivo biodistribution study in mice shows that significant volumes of nanogel and ODN accumulate in the brain following intravenous (iv) infusion of ODN synthesized in nanogel [211].

## 7. Applications of Cross-Linked Gels in CNS Drug Delivery

The central nervous system (CNS) controls the collection and analysis of peripheral information to manage the behavior of an organism. CNS conditions, such as brain tumors, neurodegenerative illnesses, and CNS injuries, are traumatic and can result in life-long disabilities [212].

**Figure 10:**
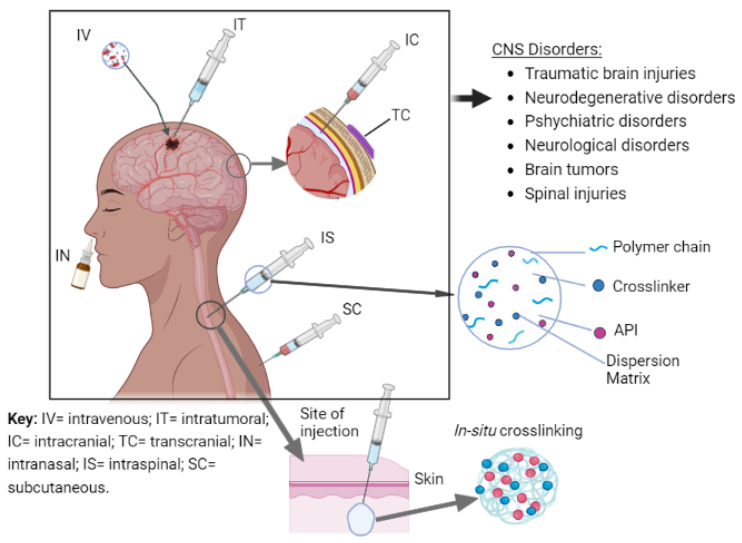
Pictorial illustration of the in-situ gel formation following the administration of drug loaded cross-linked gel for CNS conditions. Key: IN = Intranasal injection; IC = Intracranial; SC = Subcutaneous Injection, IV = Intravenous and IS = Intraspinal.

Low CNS concentrations are frequently the outcome of medication delivery via systemic routes of administration. One of the most common techniques to bypass the limitations posed by the BBB on CNS delivery is convection-enhanced delivery (CED). CED is a method that involves injecting active substances directly into the parenchyma while maintaining a positive gradient. This dilates the extracellular space and increases the volume of distribution [213]. Cross-linked gels are generally regarded as promising drug delivery systems for the central nervous system as they can be tailored to modify the release of API allowing for improved dosing intervals as well as having the ability for spatial drug delivery and extended routes of administration, as depicted in Figure 10 [214].

Injuries to the CNS can be caused by traumatic brain injury (TBI), neurodegenerative diseases, neurodevelopmental disorders, transient ischaemic attack/stroke, and brain tumors. The poor regeneration of the CNS parenchyma and scar formation at the lesion site make brain damage irreversible, resulting in long-term disability [215]. Consequently, the application of biomaterial scaffolds to fill brain lesions has grown into a field of focus. Cross-linked gels, particularly hydrogels have been proven to be ideal biomaterials for brain tissue regeneration [215]. Their admirable 3-D cross-linked polymer network with over 90% water content, in addition to their adjustable physical and chemical properties, enables hydrogels to provide a favorable microenvironment for nerve cells’ growth and proliferation. The hydrogels applied to repair CNS injuries can be administered via injectable or non-injectable routes. Depending on the site damage whether it’s the spinal area or the brain parenchyma, either injectable or non-injectable cross-linked gels may be administered [215].

### 7.1. Injectable Cross-Linked Gels

Injectable drug delivery routes for CNS delivery include the development of long-acting injectable (LAI) antipsychotics also known as depot antipsychotics. The goal of developing LAI was to maintain steady plasma drug concentrations and so lower the risk of relapse and side effects. This was following oral antipsychotic discontinuation rates of 26 to 44% and two-thirds of patients being partially non-adherent [216]. Another potentially effective injectable method of medicine delivery is intrathecal (IT) administration, which involves injecting drugs directly into the thecal sac that houses cerebrospinal fluid (CSF). This mode of administration can minimize off-target exposure and associated toxicity while achieving a high concentration of the therapeutic substance within the CNS [187].

Perispinal injection is a new technique for administering medication to the CNS. Following systemic injection, physiological obstacles prevent macromolecules from effectively entering the CNS. Perispinal injection is intended to improve drug delivery to the CNS by utilizing the cerebrospinal venous system (CSVS). The external vertebral venous plexus (EVVP), a section of the CSVS, drains the anatomic region posterior to the ligamentum flavum, where it distributes substances. The deeper venous plexuses of the CSVS can communicate with the blood in the EVVP [217].

Shatsberg et al. developed polymeric nanogels for intratumoral injection based on a polyglycerol scaffold as a new method of delivering miRNA for Glioblastoma Multiforme (GBM). Emphasis was placed on miR-34a due to its essential function key in oncogenic pathways and its capacity to limit tumor growth in GBM and other cancers. The tumor growth in the xenograft mice was remarkably inhibited as a result of the microRNA-carrying nanogels’ assistance in restoring miR-34a’s tumor suppressor function [218].

A magnetic pH and temperature-sensitive nanogel were developed for intratumoral injection. The novel magnetic pH and temperature-sensitive nanogels were linked with Cyanine-5.5 (Cy5.5)-labeled lactoferrin as a potential contrast agent for the treatment of glioma. The nanogels were intended for the pre-operative MRI diagnostic of the glioma, in which case the nanogels were found to accumulate specifically in the tumor tissues. It was observed that only the area of the rat’s brain tumor that received nanogels showed a detectable fluorescence signal, proving the biocompatibility of the nanogels. In addition, no significant harmful effects were seen in key biological processes or major organs in the rat models [219].

It was demonstrated that the biocompatibility of nanogels that mimics the cellular membrane is the key factor for effective drug delivery across the BBB. Jiang et al. formulated a cross-linked gel using azobenzene as a crosslinker and a phosphorylcholine polymer loaded with doxorubicin. They synthesized nanogels for delivery of doxorubicin to the brains of mice and indicated the superior therapeutic behaviors in the treatment of glioblastoma. They found that nanogels can pass through the BBB and exhibited a high accumulation of the payload in glioblastoma tissue due to the phosphorylcholine mimicking cellular membrane [220].

Cross-linked gels have been used to target tumor microenvironments. Some of the polymers used in cross-linking may possess tumor microenvironment targeting, scavenging, and/or altering properties [221]. Examples of such polymers include *N*-(2-hydroxypropyl)methacrylamide (HPMA) [221] and poly (propylene sulfide)120 [222]. Qian et al., developed an injectable reactive oxygen species (ROS) depletion hydrogel loaded with curcumin for TBI to promote the regeneration and recovery of neurons. Triglycerol monostearate was utilized as a crosslinker with poly (propylene sulfide) 120 (PPS 120), which is inherently a ROS quencher [222]. The hydrogel was directly injected into the wound cavity of the area of brain injury to bypass the BBB, thus enhancing drug accumulation. The DDS gelates in response to hydrogen peroxide exposure in the brain trauma microenvironment. The biodegradation of the crosslinker exposed PPS 120 to ROS, further decreasing ROS-mediated neurodegeneration [222]. Not only does the polymer enhance the PK properties of the API to improve its therapeutic efficacy, but it also elicits pharmacological effects that augment the effects of the API.

Nazemi et al. developed a dual-delivery system that delivers paclitaxel (PTX) and minocycline hydrochloride (MH), two drugs that promote tissue regeneration in a rat hemisection model of spinal cord injury. Histological evaluations revealed that the combined drug therapy reduced inflammation after seven days. In addition, after 28 days, rats given the dual-drug delivery system showed a reduction in scar tissue and an increase in neuronal regeneration. Compared to other experimental groups, animals treated with two drugs over time showed a rapid and durable improvement in their functional state [223].

The thermosensitive injectable hydrogel was developed by Li et al. for the sustained, gradual release of activin B into the striatum of mouse models with Parkinson’s disease. The hydrogel method and the intended drug release kinetics of activin B were found to be largely responsible for the significant cellular protection and clinical recovery in Parkinson’s disease mice [224]. Zhang et al. developed and prepared an injectable hydrogel made of hyaluronic acid (HA) and sodium alginate (SA) to use as the tissue scaffold for the potential treatment of traumatic brain injury [225]. According to in-vivo studies conducted, HA/SA hydrogels have shown to be the ideal scaffold for human umbilical cord-derived mesenchymal stem cells (hUC-MSC) survival and the regeneration of endogenous nerve cells, which helps TBI patients recover their neuronal functioning [225].

Donepezil (DNP) is a hydrophilic small molecule with limited BBB permeability and is associated with increased dosing frequencies and subsequent severe cholinergic side effects [5]. To mitigate this, Kang et al. formulated a hyaluronic acid hydrogel loaded with donepezil for subcutaneous injection. The hydrogel was hybridized with microstructured lipid carriers (MLCs) and human serum albumin (HSA), which was utilized to assist in the reduction in the initial burst release and sustained release of the API. The hybridization of MLCs and HSA in the hyaluronic acid hydrogel resulted in lower maximum plasma concentration (C_max_) and a longer time to reach maximum plasma concentration (T_max_). The HSA contributed to the delay in the release of DNP, leading to a significantly higher T_max_ and reduced C_max_ values, while the MLCs significantly extended the T_max_, terminal half-life (T_1/2_), and mean residence time (MRT). In addition, the hydrogel’s structural integrity remained intact after the subcutaneous injection, resulting in a prolonged lifespan of the hydrogel, as evident in the sustained donepezil release over seven days [226]. The nucleoside reverse transcriptase inhibitors (NRTIs) used in antiviral therapy are neurotoxic and ineffective in eliminating HIV-1 from the central nervous system (CNS). To combat this, Gerson et al. previously developed cationic nanogel formulations with bioactive nucleoside analogues in the active triphosphorylated form to improve targeted drug delivery and efficacy. These formulations of phosphorylated nucleoside reverse transcriptase inhibitors (NRTIs), also known as nano-NRTIs, showed rapid absorption by macrophages and efficient reduction in HIV-1 activity in the CNS, without side effects related to NTRIs mitochondrial toxicity over extended treatment periods. They went on to further test the nano-NRTIs in humanized mouse models and evaluate their neurotoxicity. Low neurotoxicity and up to 10-fold suppression of antiviral activity were reported [227].

Although it passes through BBB very poorly, methotrexate (MTX) is a crucial therapeutic in the treatment of various central nervous system malignancies. Therefore, it is anticipated that IN delivery combined with a mucoadhesive chitosan-based nanoformulation will be able to solve this issue. Ionic gelation was used to form a nanogel that contained MTX. The nanoformulation was characterized following formulation and it displayed an average particle size close to 100 nm with a zeta potential of approximately 20 mV. According to calculations, the loading efficiency and loading capacity were approximately 70 and 3, respectively. In-vivo studies showed that drug targeting efficiency and direct transport percentage for nanogel (test) and free drug solution (control) were approximately 425% and 76% and 34,842% and 99%, respectively. Therefore, as compared to the free drug solution, in-vivo experiments showed that nanogel produced considerably larger concentrations of MTX in the brain but not in plasma. Additionally, it was found that intranasal injection of the same nanogel considerably enhances the brain concentration of MTX when compared to intravenous administration [228].

To specifically treat integrin overexpressed human glioblastoma, cyclo(Arg-Gly-Asp) peptide (cRGD) decorated disulfide (SS) containing poly(vinyl alcohol) (PVA) nanogels (cRGD-SS-NGs) were developed. The PVA nanogels were developed using inverse nanoprecipitation, “click” reaction, and cRGD conjugation and their average diameter was 142 nm. Effective targeting is possible because doxorubicin (DOX) released from cRGD-SS-NGs is significantly suppressed under physiological conditions but enhanced at endosomal pH and in response to the cytoplasmic concentration of glutathione. According to in-vivo imaging and biodistribution investigations, DOX-loaded cRGD-SS-NGs are substantially more effective at targeting human U87-MG glioblastoma xenografts in nude mice. Treatment of mice with nondecorated nanogels and free DOX results in continued tumor growth; however, treatment with DOX-loaded cRGD-SS-NGs successfully inhibits tumor growth. Furthermore, due to the nanogels’ modified surface, the DOX-loaded cRGD-SS-NGs therapy also showed significantly fewer side effects [154].

Baklaushev et al. developed cisplatin-loaded nanogels for the treatment of brain cancer that was coupled with monoclonal antibodies to connexin 43 (Cx43) and brain-specific anion transporter (BSAT1) [229]. A significant reduction was observed in rats when treated with the drug-loaded targeted with nanogel system as compared with formulations [229].

Amyloid β-protein (Aβ) production of fibrils is regarded as a crucial stage in the pathogenesis of Alzheimer’s disease (AD). A promising strategy for treating AD is to prevent Aβ aggregation. Biocompatible nanogels having hydrophobic cholesterol moieties attached to a polysaccharide pullulan backbone (cholesterol-bearing pullulan, CHP) were composed. This was achieved using the self–association in the aqueous solution approach, the nanogels had a diameter of 20–30 nm. The CHP nanogels assimilated up to 6–8 Aβ -(1–42) molecules per particle and caused Aβ to shift in conformation from a random coil to α-helix- or β-sheet-rich structure, the structure caused suppression of Aβ -(1–42) aggregation [230].

On the other hand, Wang et al. developed phenytoin sodium (PHT)-loaded angiopep-2 electro responsive hydrogel nanoparticle (ANG-ERHNPs) and (PHT) -loaded non-electroresponsive hydrogel nanoparticles (ANG-PHTHNPs) to change the content of sodium 4-vinylbenzene sulfonate in the preparation formulae. The in-vivo microdialysis analysis has shown that ANG-PHT-ERHNPs not only has the characteristics of a higher distribution in the central nervous system but also have electroresponsive ability, which resulted in a strong release of nonprotein-bound PHT during seizures. The findings show that ANG-ERHNPs may transport PHT into the brain effectively and release it when epileptiform activity occurs due to sodium 4-vinylbenzene sulfonate present in the formula [231].

A summary of the aforementioned injectable cross-linked gels and their applications in CNS disorders are provided in Table 4.

### 7.2. Non-Injectable Cross-Linked Gels

Nose-to-brain drug delivery has been achieved in various studies and has resulted in multiple pharmacokinetic improvements in drug delivered to the CNS. Such an improvement was reported by Galgatte et al., [235] when they designed and optimized a mucoadhesive ion-activated in-situ gel of sumatriptan succinate in gellan gum, for the treatment of migraines. In-vitro drug release studies of the optimized in-situ gel revealed that 98.57% of the drug is released after 5 h [235]. In-vivo studies showed improved absolute bioavailability in plasma and an increased concentration of drug reaching brain tissue [235]. Similarly, Qian et al. developed an in-situ gelling thermosensitive drug delivery system using Pluronic^®^ F-127 (PF127) loaded with tacrine for the treatment of AD. The pharmacokinetic (PK) properties of the intranasal in-situ gel were compared with tacrine oral solutions in rats, and prolonged retention within the nasal cavity in comparison with the oral solution was noted. The peak plasma concentration (C_max_) and AUC in plasma and brain tissue were increased 3-fold, translating to enhanced nasal residence time, bioavailability, and brain uptake [236]. Another thermosensitive injectable hydrogel was developed by Li et al. for the sustained, gradual release of activin B into the striatum of mouse models with Parkinson’s disease (PD). The hydrogelation method and the intended drug release kinetics of activin B were found to be largely responsible for the significant cellular protection and clinical recovery in Parkinson’s disease mice [224].

Another study conducted by Qi et al. demonstrated significant antidepressant activities with low dose treatment of behavioral despair and reserpine-induced depression with genipin-thermoresponsive hydrogel system in mouse models [232]. The hydrogel was prepared using Pluronics^®^ 407 (P 407) and Pluronics^®^ 188 (P 188), with PEG 8000 as a permeation enhancer. The relative bioavailability of genipin-THS following intranasal administration increased by ~2.13-fold compared to a genipin solution administered intraperitoneally in the hippocampus [232].

Abdulla et al. [237] developed a nanoemulsion-based in-situ gel, also called emulgel, of clozapine for intranasal administration as an approach for bioavailability enhancement. Clozapine is an antipsychotic agent used in the treatment of schizophrenia [238]. It displays limited solubility and is subject to hepatic first-pass metabolism when administered orally [237,238]. In this study, clozapine-loaded nanoemulsion was dispersed in an aqueous PF127 mobile phase. The optimized formulation demonstrated a high release profile, small average globule size (<100 nm), and low viscosity [237]. Particles with small sizes (<100 nm) are a critical factor for nose-to-brain drug delivery as they produce a larger surface area, enhancing the rate of drug absorption through olfactory and trigeminal nerve terminations [237].

McCrorie et al. developed a novel drug delivery system that uses spray technology to deliver highly drug-loaded polymeric nanocrystals coated with polylactic acid-polyethylene glycol (NCPPs); viz. Etoposide and Olaparib, directly to the brain parenchyma close to the surgical resection cavity. They used pectin-based hydrogel which showed the potential to adhere to brain tissues due to the bioadhesives forces [239]. Within the brain, pectin slowly degraded over 14 days, the mice showed no signs of neurotoxicity [239]. Similarly, a poly(N-vinyl pyrrolidone)-co-acrylic acid nanogel conjugated with insulin for intranasal delivery of insulin, was developed to evaluate the biocompatibility of the nanocarrier using blood, immunogenic, and tissue testing [240]. The nanogels significantly increased the levels of insulin in the cerebral cortex, hippocampus, and olfactory bulb at 30 min and 60 min after intranasal delivery [240]. According to these findings, nanogels’ mucoadhesive properties prolonged insulin’s duration in the mucosa and hence made it easier for insulin to penetrate the mucosa.

A pregabalin-loaded emulgel was developed using a novel biofunctional agent isolated from the fruit pulp of Musa acuminata and with sodium alginate as a standard polymer using different ratios by Madhav et al. [241]. The authors studied the mechanism of drugs released from the emulgels by fitting the release data in different kinetic models such as zero order, first order, Higuchi model, Korsmeyer-Peppas, and Hixon Crowell and by also determining the R^2^ values of the release profile corresponding to each kinetic model. The Peppas Korsmeyer with Fickian diffusion were found to be the best fit models according to the release kinetics [241].

Salem et al., developed nasal nano-emulgel considered to be a well-designed system for resveratrol (RES) delivery to the brain that exhibited a significant permeation. The intranasal safety and permeation and bioavailability enhancement were established by the optimized nasal nano-emulgel. As a result, a nasal nano-emulgel is considered an ideal choice for brain targeting. According to the results obtained, this was a significant enhancement in RES absorption by the developed nasal nano emulgel. The intranasal delivery of RES-nano emulgel to rats achieved higher plasma concentration and faster absorption rate than that by oral route, according to the pharmacokinetic investigation [242].

Liposomal DNP HCl was dispersed into thiolated chitosan hydrogel and administered via the intranasal route. The in-vivo pharmacokinetic studies showed higher drug concentration in the brain for the hydrogel, compared to the tablet administered orally [243].

A summary of the aforementioned non-injectable cross-linked gels and their applications in CNS disorders are summarized in Table 5.

## 8. Conclusions and Future Perspectives

CNS disorders have long been a challenge to treat because of the selectivity and complexity of the protective anatomical and physiological barriers. In addition, the disorders themselves may limit the treatment opportunities as some disorders such as AD compromise the integrity of the CNS barriers. At present, CNS drug delivery is focused on the non-invasive routes of administration that can impart sustainable and tunable drug expulsion, with predictable release patterns, minimal systemic side effects as well as prolonged drug residence time in the CNS. Therefore, the use of cross-linked gels is impeccable for CNS drug delivery, as they provide minimal invasiveness and are capable of delivering therapeutic drug concentrations to the CNS, even when low doses are administered. Cross-linked gels offer various advantages that make them desirable molecule carriers for drugs and biomolecules to the CNS.

Despite the in-vivo and in-vitro successes of cross-linked gels for CNS drug delivery over the years, there are no marketed cross-linked gels for CNS drug delivery applications. The strict regulatory guidelines in ensuring a reproducible quality, safety and efficacy profiles are one of the major roadblocks in the way to successful clinical translation. There is a need for further studies to understand the interaction of polymers and cross-linkers with cellular and tissue systems, as well as their biocompatibility and biodegradability that would raise safety issues such as cytotoxic, immunological and inflammatory responses. From a large-scale manufacturing point of view, methods need to be well optimized to address robustness and batch-to-batch consistency, which impacts the quality and efficacy profiles. Moreover, the translation of manufacturing cross-linked gels from lab-scale to large-scale is expensive process as the manufacturing and storage of these systems would need specialized conditions such as cold chain conditions, sterilization etc. In additions to these, the lack of long-term stability studies would impact their commercial success.

Over the years, the use of nanotechnology in the development and optimization of pharmaceutical formulations has been pivotal in the enhancement of formulation stability and cytocompatibility. The authors anticipate a widespread use of nanotechnological strategies that will improve the shortcomings and broaden the spectrum of cross-linked gels for treating various CNS disorders in both research and clinical settings.

## Figures and Tables

**Table 1:** Summary of cross-linked gels and applications.

Class of Cross-Linked Gel	Advantages	Disadvantages	Applications	Ref
**Emulgel**	Thixotropic Easily spreadable Long shelf life Improved loading efficiency Great stability	Allergic reactions Poor permeability Contact dermatitis Not easily absorbed	Topical emulgel of mefenamic acid. Topical emulgel microemulsion	[34,36,37,39,40]
**Organogel**	Ease of preparation May be used for transdermal, oral, and parenteral. Non-irritating Good resistance to microbial contamination.	Lack of biocompatibility formulations. Poor stability to temperature Greasy in nature	Intraocular flunarizine hydrochloride-loaded organogel Biodegradables in-situ forming organogel.	[42,43,44,45]
**Hydrogel**	Capable of retaining a high amount of water Hydrophilicity Biocompatibility potential Controlled drug release Smart drug delivery	It may be difficult to handle It may be difficult to sterilize Usually mechanically weak.	Chemically cross-linked by glutaraldehyde for biomedical applications. Physically cross-linked hydrogel consisting of poly (acrylamide-co-acrylic acid) (PAM-co-PAA) and poly(vinyl alcohol) (PVA).	[43,45,62,63,64,65,66,67,68,69]
**Aerogel**	High porosity Low bulk density Exceptional textural features	Low mechanical strength High environmental and economic costs of operation	Incorporation of niacin/nicotinic acid and ibuprofen in an aerogel	[73,74]
**Cryogels**	Substantial pore size and porosity High water content Great pore connectivity and consistency Flexibility of preparation Economically and environmentally friendly	Insufficient retention at injection site. Injectable cryogels may cause serios side effects. Need for repeated injections Increased costs	Thermoresponsive cryogels containing oligoehylene glycol Polyacrylic acid cryogels as PH oscillatory bromate-sulphite ferrocynide processes.	[63,66,69]

**Table 2:** A summary of natural polymers, advantages, disadvantages, crosslinkers cross-linking agents or factors used and their effect.

Polymer	Advantages	Disadvantages	Cross-Linking Agent or Factor	Effect	Ref
**Chitosan**	Antioxidant, Antifungal, Anti-inflammatory, Antibacterial Non-toxic Cost-effective Easy structure modification Thermal and chemical stability Responsive to external stimuli A polycationic character that promotes fast gelling in the basic pH of normal tissues	Relatively poor mechanical and barrier properties Naturally brittle Low lipophilicity for emulsions	Vanillin *N*,*N*/-methylene bis-acrylamide (MBA) Poly (*N*-isopropyl acrylamide	Improves the balance of chitosan between affinity and insolubility in oil due to the hydrophobic methoxyphenyl group in the vanillin aromatic ring. Assisted in the adsorption of Cr6+ ions from its water solution. High antibacterial activity cotton fabrics	[80,82,136,158,159]
**Gelatin**	Non-immunogenic Non-toxic Amphoteric Non-carcinogenic Good cell adhesion, proliferation, and differentiation due to many binding sites	Thermosensitive	2-chloro-1-methylpyridinium iodide (CMPI)	Activation of carboxylic acid sodium salt under heterogeneous reaction with high water uptake ability, reasonable biodegradability, and excellent cytocompatibility	[83,85,157]
**Alginate**	Non-toxic Non-immunogenic Good adhesion Thickening and stabilizing Gel-forming and film-forming Fiber spinning Hydrophilic Cost-effective Acidic environment neutralizer Excellent hemostatic properties	Weak mechanical strength Scarcity of efficient sites for cell adhesion, thus, poor cell attachment and proliferation. Alginate gels shrink at low pH Difficulties in sterilization, handling, and storage Difficult to control the release of alginate encapsulated material due to its porosity, permeability, and degradation	Calcium ions Sodium ions	Alginate hydrogel changed weight by 10% in pure water and 90% in an isotonic solution Selective binding to G sequences of alginate and form heat-stable three-dimensional gel networks High quantity water absorption due to ion exchange	[81,83,84,89,160,161]
**Collagen**	High antigenicity	Ethical and cultural issues Inconsistency Low mechanical strength Fast degradation rate Potential toxicity due to residual catalysts or initiators	Polypropyleneimine-octa-amine dendrimers	Supports adhesion and proliferation of human corneal epithelial cells without encouraging cellular toxicity	[92,146,157,162]
**Cellulose**	Pure A high degree of porosity Good tensile strength Low immunogenicity High relative permeability to gases and liquids High retention and ion exchange capacity	Insoluble in most solvents	Citric acid	Formation of carboxylic bridges between cellulose fibril chains, thus preventing cellulose condensation during drying Improved rehydration ability, porosity, wettability, and water swelling rate	[95,96,163]
**Hyaluronic acid**	Non-immunogenic	Usefulness degraded by hyaluronidase	*N*-(3-Dimethylaminopropyl)-*N*’-ethyl carbodiimide hydrochloride (EDC) 2-chloro-1-methylpyrinium iodide (CMPI)	Faster degradation rate and smoother surfaces, lower cytotoxicity for corneal endothelial cells, and minimal inflammatory cell infiltration or foreign body reaction after implantation Facilitates intra- and inter-molecular ester bond formation between the carboxyl and hydroxyl groups of hyaluronic and exhibits better resistance against hydrolytic degradation	[131]
**Fibrin**	Abundant and simple Resistance to degradation Fast isolation from the patient’s blood. Promotes expression of proinflammatory cytokines, cell migration, cell adhesion, and phagocytosis in monocytes, macrophages, and neutrophils.	Risk of infection transmission	Transglutaminase 2 (TG2)	Enhances proinflammatory activity to surface adhered fibrinogen	[164,165]

**Table 3:** A summary of synthetic polymers, advantages, disadvantages, cross-linking agents or factors used and their effect.

Polymer	Advantages	Disadvantages	Cross-Linking Agent or Factor	Effect	Ref
**PEG**	Amphiphilic High swelling index Good gelation properties Low immunogenicity	Low cell adhesion Poor cell affinity Reduced cellular response	Glutaraldehyde	Improved water flux and porosity Decreased swelling and solute diffusion through membranes Enhance water permeability due to hydroxyl hydrophilic functional group in GLA	[81,112,113,166]
**PVA**	Good thermal stability Good mechanical strength Excellent film membrane properties Viscoelastic hydrophilic non-toxic pH stable	Does not support cell proliferation and adhesion Limited elasticity and hydrophilicity	Citric acid	Uniformly distributed membrane roughness, homogeneous films, enhanced adhesion, and strength properties with good stability.	[112,116,117,167]
**PVP**	Low cytotoxicity Hydrophilic Excellent adsorption and adhesion Good thermal stability and miscibility Excellent wetting properties Rapid swelling Excellent film;	Weak mechanical properties Thermal instability	*N*,*N*′-methylene-bisacrylamide	Improved drug, vaccine, and peptides encapsulation	[74,168,169]
**CMC**	Hydrophilic Abundant Cost-effective Environmentally friendly	Low mechanical strength Low gelation properties	Glutaraldehyde	Improved mechanical properties due to covalent bonds formed using acidic catalysis. Improved tensile strength and elastic modulus.	[67,122,124,170]
**pNIPAAM**	Hydrophilic Good mechanical properties Temperature-responsive	Thermal instability Potential phase separation Monomers and crosslinkers use are mostly non-biodegradability and not biocompatibility, which may lead to toxic, carcinogenic, and teratogenic effects.	*N*,*N*′-methylene-bisacrylamide	The surface morphology of silicon wafers became thick, rough and thermo-responsive	[123,168,171]
**Pluronic^®^**	Amphiphilic High biocompatibility Stabilizer Retention agent	Thermosensitive Weak mechanical strength at low concentrations Lack of mucoadhesion Lower gelation temperature at high concentrations	*N*,*N*′-methylene-bisacrylamide	Improved hydrophilic properties for the solubility of poorly soluble drug olanzapine Improved drug release in both acidic and basic pH Improved safety and biocompatibility	[127,128,130,172]

**Table 4:** Summary of injectable cross-linked gels in CNS disorders.

ROA	Polymer	Crosslinker	API	Disease	In-Vivo/In-Vitro Model	In-Vivo/In-Vitro Findings	Ref
**IV**	PEG and PEI	Carbonyldiimidazole	Oligonucleotides	Neurodegenerative disorders	Mice	Better brain targeting with a 15-fold increase in accumulation of the drug in the brain and a 2-fold decrease in liver and spleen accumulation	[211]
**IT**	Polyglycerols	Disulfide	MicroRNA therapeutics	Glioblastoma Multiforme	Mice	Significantly inhibited tumor growth. Downregulation of miR-34a target genes, which plays key roles in the regulation of apoptosis and cell cycle arrest	[218]
**IV**	Phosphorylcholine	Azobenzene-contained crosslinker	Doxorubicin	Glioblastoma	Mice	Favorable biocompatibility and long-circulating property in blood Significantly stronger glioblastoma inhibition effect.	[219]
**IV**	Poly(*N*-isopropyl acrylamide-*co*-acrylic acid)	carbodiimide hydrochloride and *N*-hydroxysulfosuccinimide	Lactoferrin	Glioma	Rats	Highly sensitive and specific MR/fluorescence imaging	[220]
**IT**	Poly (propylene sulfide) 120	Triglycerol monostearate	Curcumin	TBI	Mice	Enhanced brain drug accumulation resulting in improved regeneration and recovery of neurons	[222]
**IS**	Alginate	Calcium D-gluconate monohydrate	Paclitaxel (PTX) and Minocycline hydrochloride (MH)	Spinal cord injury (SCI)	Wistar rats	Increased neuronal regeneration after 28 days. Reduced inflammation after 7 days	[223]
**IC**	PNIPAAm	poly (amidoamine)	Activin B	PD	Male C57BL/6J mice	Prolonged release of activin B of around 5 weeks	[224]
**IC**	Sodium alginate and hyaluronic acid	Calcium carbonate (CaCO^3^)	Human umbilical cord mesenchymal stem cells (hUC-MSCs)	Traumatic brain injury and stem cell tissue engineering	Sprague Dawley Rats	Enhanced regeneration of endogenous nerve cells. Protected the injected hUC-MSCs	[225]
**SC**	Hyaluronic acid	-	Donepezil	AD	Rats	Increased drug T1/2, reduced Cmax value, and sustained drug release over 7 days	[226]
**IC**	PEG and Polyethyleneimine (PEI)	1,1′-carbonyldiimidazole	Zidovudine (AZT)	HIV-1	Mice	Low neurotoxicity and improved antiviral suppression	[227]
**IC**	Poly(ethylene glycol)-b-poly(methacrylic acid) deblock copolymer	1,2-ethylenedia-mine, 1-(3-dimethylaminopropyl)-3-ethylcarbodiimide hydrochloride	Cisplatin	Intracranial gliomas	Wistar rats	Enhanced inhibition of tumor growth and increased the life span of the animals	[229]
**IV**	2-Dimethylamino ethyl methacrylate	*N*,*N*′-methylene bisacrylamide	Phenytoin sodium	Epilepsy	Male Sprague-Dawley rats	Higher distribution in the central nervous system	[231]
**IP**	Pluronics^®^ 407 (P 407), Pluronics^®^ 188 (P 188)	-	Genipin	Depression	Male ICR mice	High drug release rate and improved antidepressant-like activities	[232]
**IV**	PVA	Carbodiimide	Doxorubicin	Integrin overexpressed human glioblastoma	Nude mice	Improved tumor targeting, tumor growth inhibition, and reduced side effects	[233]
**IN**	Chitosan	Polyanionic pentasodium triphosphate	Methotrexate	Brain tumor	Sprague Dawley Rats	Up to a 10-fold increase in brain concentrations of methotrexate compared to free drug.	[234]

Key: ROA = Route of Administration; IN = Intranasal injection; IT = Intratumoral; IC = Intracranial; IP = intraperitoneal, SC = Subcutaneous Injection, IV = Intravenous and IS = Intraspinal.

**Table 5:** Summary of non-injectable cross-linked gels in CNS disorder.

ROA	Polymer Used	Cross-Linking Agent/Factor Used	API/Agent Delivered	Human Disease	In-Vivo/In-Vitro Model	In-Vivo/In-Vitro Findings	Ref
**IN**	Gellan gum	Heat	Sumatriptan succinate	Headaches	Sprague-Dawley rats	Improved brain targeting and bioavailability	[235]
**IN**	PF-127	Heat	Tacrine	AD	Rats	Increased nasal residence time, improved bioavailability, and enhanced brain uptake	[236]
**IN**	PF-127 and PF-68	Heat	Clozapine	Schizophrenia	Dialysis bag technique	Enhanced in-vitro drug release	[237]
**IC**	Pectin and poly(ethylene glycol)-block-polylactic acid (PEG-b-PLA)	Ca^2+^	Olaparib	Brain tumor	Mice	High drug loading, improved in-vitro stability, and drug release over prolonged periods	[239]
**IN**	Poly(N-vinyl pyrrolidone)-co-acrylic acid	1-ethyl-3-[3- dimethylaminopropyl] carbodiimide hydrochloride	Insulin	AD	Male C57BL/6J (B6) mice	Non-immunogenic response of the nasal mucosa. Enhanced distribution of insulin to different brain areas.	[240]
**TC**	Sodium Alginate	Aqueous solvent	Pregabalin	Epilepsy	Dialysis membrane	Faster drug release, biodegradable, biocompatible, non-toxic, non-irritant, and no reaction on the skin were observed.	[241]
**IN**	Carbopol 934 and Pluronics^®^ 407	Potassium persulfate	Resveratrol	Brain tumors	Wistar albino rats	Good drug release properties. Safe and tolerable to the nasal mucosa	[242]
**IN**	Chitosan	Glutaraldehyde	Liposomal donepezil HCl	AD	New Zealand white rabbits	Significant increase in blood concentration and brain content of the API, compared to the oral tablets	[243]

Key: ROA = Route of Administration; IN = Intranasal and TC = Transcranial.

## Data Availability

Not applicable.

## References

[1] O’Loinsigh E., Bose A. (2019). Regulatory Considerations for the Use of Biomarkers and Personalized Medicine in CNS Drug Development: A European Perspective. Handbook of Behavioral Neuroscience.

[2] Li G., Shao K., Umeshappa C.S. (2019). Recent Progress in Blood-Brain Barrier Transportation Research. Brain Targeted Drug Delivery System.

[3] Gitler A.D., Dhillon P., Shorter J. (2017). Neurodegenerative Disease: Models, Mechanisms, and a New Hope. Dis. Model. Mech..

[4] Vieira A., Filho M., Chaves S.N., Martins W.R., Tolentino G.P., De R., Pereira C., Homem P., De Farias G.L., Fischer B.L. (2020). Progressive Resistance Training Improves Bradykinesia, Motor Symptoms and Functional Performance in Patients with Parkinson’s Disease. Clin. Interv. Aging.

[5] Witika B.A., Poka M.S., Demana P.H., Matafwali S.K., Melamane S., Malungelo Khamanga S.M., Makoni P.A. (2022). Lipid-Based Nanocarriers for Neurological Disorders: A Review of the State-of-the-Art and Therapeutic Success to Date. Pharmaceutics.

[6] Taylor J.P., Brown R.H., Cleveland D.W. (2016). Decoding ALS: From Genes to Mechanism. Nature.

[7] National Department of Health South Africa Essential Drugs Programme (2019). Hospital Level (Adults) Standard Treatment Guidelines and Essential Medicines List.

[8] Alahmari A. (2021). Blood-Brain Barrier Overview: Structural and Functional Correlation. Neural Plast..

[9] Rathod H., Mehta D., Author C., Rathod H.J., Mehta D.P. (2015). A Review on Pharmaceutical Gel. Int. J. Pharm. Sci..

[10] Soni K.S., Desale S.S., Bronich T.K. (2016). Nanogels: An Overview of Properties, Biomedical Applications and Obstacles to Clinical Translation. J. Control. Release.

[11] Keskin D., Zu G., Forson A.M., Tromp L., Sjollema J., van Rijn P. (2021). Nanogels: A Novel Approach in Antimicrobial Delivery Systems and Antimicrobial Coatings. Bioact. Mater..

[12] Mauri E., Giannitelli S.M., Trombetta M., Rainer A. (2021). Synthesis of Nanogels: Current Trends and Future Outlook. Gels.

[13] Gawdi R., Shumway K.R., Emmady P.D. Physiology, Blood Brain Barrier. http://www.ncbi.nlm.nih.gov/pubmed/32491653.

[14] Ndemazie N.B., Inkoom A., Morfaw E.F., Smith T., Aghimien M., Ebesoh D., Agyare E. (2022). Multi-Disciplinary Approach for Drug and Gene Delivery Systems to the Brain. AAPS PharmSciTech.

[15] Begley D.J. (2004). Delivery of Therapeutic Agents to the Central Nervous System: The Problems and the Possibilities. Pharmacol. Ther..

[16] Upton D.H., Ung C., George S.M., Tsoli M., Kavallaris M., Ziegler D.S. (2022). Challenges and Opportunities to Penetrate the Blood-Brain Barrier for Brain Cancer Therapy. Theranostics.

[17] Girardin F. (2006). Membrane Transporter Proteins: A Challenge for CNS Drug Development. Dialogues Clin. Neurosci..

[18] Tumani H., Huss A., Bachhuber F. (2018). The Cerebrospinal Fluid and Barriers—Anatomic and Physiologic Considerations. Handbook of Clinical Neurology.

[19] Engelhardt B., Sorokin L. (2009). The Blood-Brain and the Blood-Cerebrospinal Fluid Barriers: Function and Dysfunction. Semin. Immunopathol..

[20] Strazielle N., Ghersi-Egea J.F. (2015). Efflux Transporters in Blood-Brain Interfaces of the Developing Brain. Front. Neurosci..

[21] Agarwal S., Hartz A.M.S., Elmquist W.F., Bauer B. (2011). Breast Cancer Resistance Protein and P-Glycoprotein in Brain Cancer: Two Gatekeepers Team Up. Curr. Pharm. Des..

[22] Löscher W., Potschka H., Sisodiya S.M., Vezzani A. (2020). Drug Resistance in Epilepsy: Clinical Impact, Potential Mechanisms, and New Innovative Treatment Options. Pharmacol. Rev..

[23] Tishler D.M., Weinberg K.I., Hinton D.R., Barbaro N., Annett G.M., Raffel C. (1995). MDR1 Gene Expression in Brain of Patients with Medically Intractable Epilepsy. Epilepsia.

[24] Sakata S., Fujiwara M., Ohtsuka K., Kamma H., Nagane M., Sakamoto A., Fujioka Y. (2011). ATP-Binding Cassette Transporters in Primary Central Nervous System Lymphoma: Decreased Expression of MDR1 P-Glycoprotein and Breast Cancer Resistance Protein in Tumor Capillary Endothelial Cells. Oncol. Rep..

[25] Ginguené C., Champier J., Maallem S., Strazielle N., Jouvet A., Fèvre-Montange M., Ghersi-Egea J.F. (2010). P-glycoprotein (ABCB1) and Breast Cancer Resistance Protein (ABCG2) Localize in the Microvessels Forming the Blood-Tumor Barrier in Ependymomas. Brain Pathol..

[26] Vendel E., Rottschäfer V., de Lange E.C.M. (2020). The 3D Brain Unit Network Model to Study Spatial Brain Drug Exposure under Healthy and Pathological Conditions. Pharm. Res..

[27] Drouin-Ouellet J., Sawiak S.J., Cisbani G., Lagacé M., Kuan W.-L., Saint-Pierre M., Dury R.J., Alata W., St-Amour I., Mason S.L. (2015). Cerebrovascular and Blood-Brain Barrier Impairments in Huntington’s Disease: Potential Implications for Its Pathophysiology. Ann. Neurol..

[28] Arvanitis C.D., Ferraro G.B., Jain R.K. (2020). The Blood–Brain Barrier and Blood–Tumour Barrier in Brain Tumours and Metastases. Nat. Rev. Cancer.

[29] Mayhan W.G. (1999). VEGF Increases Permeability of the Blood-Brain Barrier via a Nitric Oxide Synthase/CGMP-Dependent Pathway. Am. J. Physiol. Physiol..

[30] Fukumura D., Jain R.K. (1998). Role of Nitric Oxide in Angiogenesis and Microcirculation in Tumors. Cancer Metastasis Rev..

[31] Jablonski M.R., Markandaiah S.S., Jacob D., Meng N.J., Li K., Gennaro V., Lepore A.C., Trotti D., Pasinelli P. (2014). Inhibiting Drug Efflux Transporters Improves Efficacy of ALS Therapeutics. Ann. Clin. Transl. Neurol..

[32] Li J., Zheng M., Shimoni O., Banks W.A., Bush A.I., Gamble J.R., Shi B. (2021). Development of Novel Therapeutics Targeting the Blood-Brain Barrier: From Barrier to Carrier. Adv. Sci..

[33] Nance E., Pun S.H., Saigal R., Sellers D.L. (2022). Drug Delivery to the Central Nervous System. Nat. Rev. Mater..

[34] Sabalingam S., Siriwardhene M.A. (2022). A Review on Emerging Applications of Emulgel as Topical Drug Delivery System. World J. Adv. Res. Rev..

[35] Sharma V., Nayak S.K., Paul S.R., Choudhary B., Ray S.S., Pal K. (2018). Emulgels. Polym. Gels.

[36] Talat M., Zaman M., Khan R., Jamshaid M., Akhtar M., Mirza A.Z. (2021). Emulgel: An Effective Drug Delivery System. Drug Dev. Ind. Pharm..

[37] Verma A., Jain A., Tiwari A., Jain S.K. (2018). Emulgels: Application Potential in Drug Delivery. Functional Biopolymers.

[38] Redkar M.R., Patil S.V., Rukari T.G. (2019). (PDF) Emulgel: A Modern Tool For Topical Drug Delivery. World J. Pharm. Res..

[39] Sreevidya V.S. (2019). An Overview on Emulgel. Int. J. Pharm. Phytopharm. Res..

[40] Ashara K., Soniwala M., Shah K. (2016). Review Article Emulgel: A Novel Drug Delivery System. J. Pakistan Assoc. Dermatol..

[41] Lampp L., Rogozhnikova O.Y., Trukhin D.V., Tormyshev V.M., Bowman M.K., Devasahayam N., Krishna M.C., Mäder K., Imming P. (2019). A Radical Containing Injectable In-Situ-Oleogel and Emulgel for Prolonged in-Vivo Oxygen Measurements with CW EPR. Free Radic. Biol. Med..

[42] Vintiloiu A., Leroux J.C. (2008). Organogels and Their Use in Drug Delivery—A Review. J. Control. Release.

[43] Mujawar N.K., Ghatage S.L., Yeligar V.C. (2014). Organogel: Factors And Its Importance. Int. J. Biol. Chem. Sci..

[44] Esposito C.L., Kirilov P., Roullin V.G. (2018). Organogels, Promising Drug Delivery Systems: An Update of State-of-the-Art and Recent Applications. J. Control. Release.

[45] Das J., Bhattacharjee B., Dutta J.J. (2021). Tirna Paul ORGANOGEL: An Ideal Drug Delivery Carrier. World J. Pharm. Res..

[46] Wang D., Zhao J., Liu X., Sun F., Zhou Y., Teng L., Li Y. (2014). Parenteral Thermo-Sensitive Organogel for Schizophrenia Therapy, in Vitro and in Vivo Evaluation. Eur. J. Pharm. Sci..

[47] Memic A., Colombani T., Eggermont L.J., Rezaeeyazdi M., Steingold J., Rogers Z.J., Joshi Navare K., Mohammed H.S., Bencherif S.A., Memic A. (2019). Latest Advances in Cryogel Technology for Biomedical Applications. Adv. Ther..

[48] Eggermont L.J., Rogers Z.J., Colombani T., Memic A., Bencherif S.A. (2020). Injectable Cryogels for Biomedical Applications. Trends Biotechnol..

[49] Razavi M., Qiao Y., Thakor A.S. (2019). Three-Dimensional Cryogels for Biomedical Applications. J. Biomed. Mater. Res. A.

[50] Lozinsky V.I. (2018). Cryostructuring of Polymeric Systems. 50. Cryogels and Cryotropic Gel-Formation: Terms and Definitions. Gels.

[51] Savina I.N., Zoughaib M., Yergeshov A.A. (2021). Design and Assessment of Biodegradable Macroporous Cryogels as Advanced Tissue Engineering and Drug Carrying Materials. Gels.

[52] Jones L.O., Williams L., Boam T., Kalmet M., Oguike C., Hatton F.L. (2021). Cryogels: Recent Applications in 3D-Bioprinting, Injectable Cryogels, Drug Delivery, and Wound Healing. Beilstein J. Org. Chem..

[53] Zhang X., Liu K., Liu J., Ding Y., Li W., Zhang A. (2020). Thermoresponsive Cryogels from Dendronized Interpenetrating Polymer Network Showing Dual-Shape Memory. Eur. Polym. J..

[54] Klouda L., Mikos A.G. (2008). Thermoresponsive Hydrogels in Biomedical Applications. Eur. J. Pharm. Biopharm..

[55] Pasparakis G., Vamvakaki M. (2011). Multiresponsive Polymers: Nano-Sized Assemblies, Stimuli-Sensitive Gels and Smart Surfaces. Polym. Chem..

[56] Bilici C., Karayel S., Demir T.T., Okay O. (2010). Self-Oscillating PH-Responsive Cryogels as Possible Candidates of Soft Materials for Generating Mechanical Energy. J. Appl. Polym. Sci..

[57] García-González C.A., Sosnik A., Kalmár J., De Marco I., Erkey C., Concheiro A., Alvarez-Lorenzo C. (2021). Aerogels in Drug Delivery: From Design to Application. J. Control. Release.

[58] García-González C.A., Budtova T., Durães L., Erkey C., Del Gaudio P., Gurikov P., Koebel M., Liebner F., Neagu M., Smirnova I. (2019). An Opinion Paper on Aerogels for Biomedical and Environmental Applications. Molecules.

[59] Maleki H., Durães L., García-González C.A., del Gaudio P., Portugal A., Mahmoudi M. (2016). Synthesis and Biomedical Applications of Aerogels: Possibilities and Challenges. Adv. Colloid Interface Sci..

[60] Michel A., Matthias K., Nicholas L. (2011). Aerogels Handbook.

[61] Ferreira-Gonçalves T., Constantin C., Neagu M., Reis C.P., Sabri F., Simón-Vázquez R. (2021). Safety and Efficacy Assessment of Aerogels for Biomedical Applications. Biomed. Pharmacother..

[62] Wang W., Narain R., Zeng H. (2020). Hydrogels. Polymer Science and Nanotechnology.

[63] Varaprasad K., Raghavendra G.M., Jayaramudu T., Yallapu M.M., Sadiku R. (2017). A Mini Review on Hydrogels Classification and Recent Developments in Miscellaneous Applications. Mater. Sci. Eng. C.

[64] Sharma S., Tiwari S. (2020). A Review on Biomacromolecular Hydrogel Classification and Its Applications. Int. J. Biol. Macromol..

[65] Garg S., Garg A. (2016). Hydrogel: Classification, Properties, Preparation and Technical Features. Asian J. Biomater. Res..

[66] Ahmed E.M. (2015). Hydrogel: Preparation, Characterization, and Applications: A Review. J. Adv. Res..

[67] Ghasemiyeh P., Mohammadi-Samani S. (2019). Hydrogels as Drug Delivery Systems; Pros and Cons. Trends Pharm. Sci..

[68] Peppas N.A., Hoffman A.S. (2020). Hydrogels. Biomaterials Science.

[69] Narayanaswamy R., Torchilin V.P. (2019). Hydrogels and Their Applications in Targeted Drug Delivery. Molecules.

[70] Jalalvandi E., Cabral J., Hanton L.R., Moratti S.C. (2016). Cyclodextrin-Polyhydrazine Degradable Gels for Hydrophobic Drug Delivery. Mater. Sci. Eng. C Mater. Biol. Appl..

[71] Chen G., Li J., Cai Y., Zhan J., Gao J., Song M., Shi Y., Yang Z. (2017). A Glycyrrhetinic Acid-Modified Curcumin Supramolecular Hydrogel for Liver Tumor Targeting Therapy. Sci. Rep..

[72] Akiyoshi K., Taniguchi I., Fukui H., Sunamoto J. (1996). Hydrogel Nanoparticle Formed by Self-Assembly of Hydrophobized Polysaccharide. Stabilization of Adriamycin by Complexation. Eur. J. Pharm. Biopharm..

[73] Gong Z., Zhang G., Zeng X., Li J., Li G., Huang W., Sun R., Wong C. (2016). High-Strength, Tough, Fatigue Resistant, and Self-Healing Hydrogel Based on Dual Physically Cross-Linked Network. ACS Appl. Mater. Interfaces.

[74] Capanema N.S.V., Mansur A.A.P., de Jesus A.C., Carvalho S.M., de Oliveira L.C., Mansur H.S. (2018). Superabsorbent Crosslinked Carboxymethyl Cellulose-PEG Hydrogels for Potential Wound Dressing Applications. Int. J. Biol. Macromol..

[75] Wang K., Zhang X., Li C., Sun X., Meng Q., Ma Y., Wei Z. (2015). Chemically Crosslinked Hydrogel Film Leads to Integrated Flexible Supercapacitors with Superior Performance. Adv. Mater..

[76] Ahmad Z., Salman S., Khan S.A., Amin A., Rahman Z.U., Al-Ghamdi Y.O., Akhtar K., Bakhsh E.M., Khan S.B., Ahmad Z. (2022). Versatility of Hydrogels: From Synthetic Strategies, Classification, and Properties to Biomedical Applications. Gels.

[77] Onaciu A., Munteanu R.A., Moldovan A.I., Moldovan C.S., Berindan-Neagoe I. (2019). Hydrogels Based Drug Delivery Synthesis, Characterization and Administration. Pharmaceutics.

[78] Caillol S. (2020). Molecules Special Issue “Natural Polymers and Biopolymers II”. Molecules.

[79] Singhal R., Gupta K. (2016). A Review: Tailor-Made Hydrogel Structures (Classifications and Synthesis Parameters). Polym. Plast. Technol. Eng..

[80] Do N.H.N., Truong Q.T., Le P.K., Ha A.C. (2021). Journal Pre-Proof Recent Developments in Chitosan Hydrogels Carrying Natural Bioactive Compounds. Carbohydr. Polym..

[81] Radulescu D.M., Neacsu I.A., Grumezescu A.M., Andronescu E. (2022). New Insights of Scaffolds Based on Hydrogels in Tissue Engineering. Polymers.

[82] Andrade J., Alonso J.M., Sáez V., Cid S.B., Moreno-benítez I., Larrauri B., González R.P., Vilas-vilela J.L., Pérez-álvarez L. (2022). Self-Healing, Antibacterial and Anti-Inflammatory Chitosan-PEG Hydrogels for Ulcerated Skin Wound Healing and Drug Delivery. Biomater. Adv..

[83] Ramdhan T., Ching S.H., Prakash S., Bhandari B. (2020). Physical and Mechanical Properties of Alginate Based Composite Gels. Trends Food Sci. Technol..

[84] Phatchayawat P.P., Khamkeaw A., Yodmuang S., Phisalaphong M. (2022). 3D Bacterial Cellulose-Chitosan-Alginate-Gelatin Hydrogel Scaffold for Cartilage Tissue Engineering. Biochem. Eng. J..

[85] Jaipan P., Nguyen A., Narayan R.J. (2017). Biomaterials for 3D Cell Biology Prospective Article. MRS Commun..

[86] Carmona S., Mellado C., Mel M., Aguayo C., Fern K. (2022). Biomaterials Advances Novel and Effective Hemostats Based on Graphene Oxide-Polymer Aerogels: In Vitro and in Vivo Evaluation. Biomater. Adv..

[87] Kharkar P.M., Kiick K.L., Kloxin A.M. (2013). Designing Degradable Hydrogels for Orthogonal Control of Cell Microenvironments. Chem. Soc. Rev..

[88] Bulut E., Şanlı O. (2016). Novel Ionically Crosslinked Acrylamide-Grafted Poly(Vinyl Alcohol)/Sodium Alginate/Sodium Carboxymethyl Cellulose PH-Sensitive Microspheres for Delivery of Alzheimer’s Drug Donepezil Hydrochloride: Preparation and Optimization of Release Conditions. Artif. Cells Nanomed. Biotechnol..

[89] Bogdanova L.R., Zelenikhin P.V., Makarova A.O., Zueva O.S., Salnikov V.V., Zuev Y.F., Ilinskaya O.N. (2022). Alginate-Based Hydrogel as Delivery System for Therapeutic Bacterial RNase. Polymers.

[90] Liu K., Wiendels M., Yuan H., Ruan C., Kouwer P.H.J. (2022). Cell-Matrix Reciprocity in 3D Culture Models with Nonlinear Elasticity. Bioact. Mater..

[91] Ho T.-C., Chang C.-C., Chan H.-P., Chung T.-W., Shu C.-W., Chuang K.-P., Duh T.-H., Yang M.-H., Tyan Y.-C. (2022). Hydrogels: Properties and Applications in Biomedicine. Molecules.

[92] Promoteur E. (2022). In Silico Model for Gel Aspiration-Ejection (GAE) Process in the Context of Clinical Peripheral Nerve Repair. Master’s Thesis.

[93] Kang H., Liu R., Huang Y. (2016). Cellulose-Based Gels. Macromol. Chem. Phys..

[94] Zhang H., Wang Y., Chen W., Chen Y. (2022). Thiol-Ene Crosslinked Cellulose-Based Gel Polymer Electrolyte with Good Structural Integrity for High Cycling Performance Lithium-Metal Battery. Res. Sq..

[95] Ullah H., Santos H.A., Khan T. (2016). Applications of Bacterial Cellulose in Food, Cosmetics and Drug Delivery. Cellulose.

[96] Das S., Ghosh B., Sarkar K. (2022). Nanocellulose as Sustainable Biomaterials for Drug Delivery. Sens. Int..

[97] Dovedytis M., Liu Z.J., Bartlett S. (2020). Hyaluronic Acid and Its Biomedical Applications: A Review. Eng. Regen..

[98] Nikjoo D., van der Zwaan I., Brülls M., Tehler U., Frenning G. (2021). Hyaluronic Acid Hydrogels for Controlled Pulmonary Drug Delivery—A Particle Engineering Approach. Pharmaceutics.

[99] Schneider-Barthold C., Baganz S., Wilhelmi M., Scheper T., Pepelanova I. (2016). Hydrogels Based on Collagen and Fibrin—Frontiers and Applications. BioNanoMaterials.

[100] Murphy K.C., Whitehead J., Zhou D., Ho S.S., Leach J.K. (2017). Engineering Fibrin Hydrogels to Promote the Wound Healing Potential of Mesenchymal Stem Cell Spheroids. Acta Biomater..

[101] Ahearne M., Buckley C.T., Kelly D.J. (2011). A Growth Factor Delivery System for Chondrogenic Induction of Infrapatellar Fat Pad-Derived Stem Cells in Fibrin Hydrogels. Biotechnol. Appl. Biochem..

[102] Gandhi J.K., Manzar Z., Bachman L.A., Andrews-Pfannkoch C., Knudsen T., Hill M., Schmidt H., Iezzi R., Pulido J.S., Marmorstein A.D. (2018). Fibrin Hydrogels as a Xenofree and Rapidly Degradable Support for Transplantation of Retinal Pigment Epithelium Monolayers. Acta Biomater..

[103] Tanaka R., Saito Y., Fujiwara Y., Jo J., Tabata Y. (2019). Preparation of Fibrin Hydrogels to Promote the Recruitment of Anti-Inflammatory Macrophages. Acta Biomater..

[104] Aswathy S.H., Narendrakumar U., Manjubala I. (2020). Commercial Hydrogels for Biomedical Applications. Heliyon.

[105] Hassan T., Zhou C., Saeed S. (2021). Polymers, An Infrangible Part of Our Life. J. Islam. Med. Dent. Coll..

[106] Bi X., Liang A. (2016). In Situ-Forming Cross-linking Hydrogel Systems: Chemistry and Biomedical Applications. Emerg. Concepts Anal. Appl. Hydrogels.

[107] Maitra J., Shukla V.K. (2014). Cross-Linking in Hydrogels—A Review. Am. J. Polym. Sci..

[108] Priya V.S.V., Roy H.K., Jyothi N., Prasanthi N.L. (2016). Polymers in Drug Delivery Technology, Types of Polymers and Applications. Sch. Acad. J. Pharm..

[109] Larrañeta E., Stewart S., Ervine M., Al-Kasasbeh R., Donnelly R.F. (2018). Functional Biomaterials Hydrogels for Hydrophobic Drug Delivery. Classification, Synthesis and Applications. J. Funct. Biomater..

[110] Kfoury M., Landy D., Fourmentin S. (2022). Combination of DES and Macrocyclic Host Molecules: Review and Perspectives. Curr. Opin. Green Sustain. Chem..

[111] Nafo Id W., Al-Mayah A. (2020). Mechanical Characterization of PVA Hydrogels’ Rate-Dependent Response Using Multi-Axial Loading. PLoS ONE.

[112] Fatima F., Singh V. (2022). Materials Today: Proceedings Assessment of Antibacterial Properties of Electrospun Fish Collagen/Poly (Vinyl) Alcohol Nanofibers with/Biosurfactant Rhamnolipid. Mater. Today Proc..

[113] Husain M.S.B., Gupta A., Alashwal B.Y., Sharma S. (2018). Synthesis of PVA/PVP Based Hydrogel for Biomedical Applications: A Review. Energy Sources, Part A Recover. Util. Environ. Eff..

[114] Contardi M., Kossyvaki D., Picone P., Summa M., Guo X., Heredia-Guerrero J.A., Giacomazza D., Carzino R., Goldoni L., Scoponi G. (2021). Electrospun Polyvinylpyrrolidone (PVP) Hydrogels Containing Hydroxycinnamic Acid Derivatives as Potential Wound Dressings. Chem. Eng. J..

[115] Awasthi R., Manchanda S., Das P., Velu V., Malipeddi H., Pabreja K., Pinto T.D.J.A., Gupta G., Dua K. (2018). Poly(Vinylpyrrolidone). Engineering of Biomaterials for Drug Delivery Systems.

[116] Gibas I., Janik H. (2010). Review: Synthetic Polymer Hydrogels for Biomedical Applications. Chem. Chem. Technol..

[117] Zhang H., Wu X., Qin Z., Sun X., Zhang H., Yu Q., Yao M., He S., Dong X., Yao F. (2020). Dual Physically Cross-Linked Carboxymethyl Cellulose-Based Hydrogel with High Stretchability and Toughness as Sensitive Strain Sensors. Cellulose.

[118] Long L., Li F., Shu M., Zhang C., Weng Y. (2019). Materials Fabrication and Application of Carboxymethyl Cellulose-Carbon Nanotube Aerogels. Materials.

[119] Wei G., Yang D., Zhang T., Yue X., Qiu F. (2020). Thermal-Responsive PNIPAm-Acrylic/Ag NRs Hybrid Hydrogel with Atmospheric Window Full-Wavelength Thermal Management for Smart Windows. Sol. Energy Mater. Sol. Cells.

[120] Peppas N.A., Hilt J.Z., Khademhosseini A., Langer R. (2006). Hydrogels in Biology and Medicine: From Molecular Principles to Bionanotechnology. Adv. Mater..

[121] Hua L., Xie M., Jian Y., Wu B., Chen C., Zhao C. (2019). Multiple-Responsive and Amphibious Hydrogel Actuator Based on Asymmetric UCST-Type Volume Phase Transition. ACS Appl. Mater. Interfaces.

[122] Mourey T.H., Schunk T.C. (2019). Synthetic Polymers.

[123] Yu J., Qiu H., Yin S., Wang H., Li Y. (2021). Polymeric Drug Delivery System Based on Pluronics for Cancer Treatment. Molecules.

[124] Mahajan H.S., Jadhao V.D. (2022). Pullulan and Pluronic F-127 Based in Situ Gel System for Intranasal Delivery: Development, in Vitro and in Vivo Evaluation. J. Bioact. Compat. Polym..

[125] Huang J., Wang Z., Krishna S., Hu Q., Xuan M., Xie H. (2020). Environment-Sensitive Hydrogels as Potential Drug Delivery Systems for the Treatment of Periodontitis. Mater. Express.

[126] Gegel N.O., Shipovskaya A.B., Khaptsev Z.Y., Radionov R.V., Belyaeva A.A., Kharlamov V.N., Gegel N.O., Shipovskaya A.B., Khaptsev Z.Y., Radionov R.V. (2022). Thermosensitive Chitosan-Containing Hydrogels: Their Formation, Properties, Antibacterial Activity, and Veterinary Usage. Gels.

[127] Boonlai W., Tantishaiyakul V., Hirun N., Sangfai T., Suknuntha K. (2018). Thermosensitive Poloxamer 407/Poly(Acrylic Acid) Hydrogels with Potential Application as Injectable Drug Delivery System. AAPS PharmSciTech.

[128] Parhi R. (2017). Cross-Linked Hydrogel for Pharmaceutical Applications: A Review. Adv. Pharm. Bull..

[129] Bahrani S., Aslani R., Hashemi S.A., Mousavi S.M., Ghaedi M. (2021). Introduction to Molecularly Imprinted Polymer. Interface Sci. Technol..

[130] Alavarse A.C., Frachini E.C.G., da Silva R.L.C.G., Lima V.H., Shavandi A., Petri D.F.S. (2022). Crosslinkers for Polysaccharides and Proteins: Synthesis Conditions, Mechanisms, and Crosslinking Efficiency, a Review. Int. J. Biol. Macromol..

[131] Zafar S., Hanif M., Azeem M., Mahmood K., Gondal S.A. (2021). Role of Crosslinkers for Synthesizing Biocompatible, Biodegradable and Mechanically Strong Hydrogels with Desired Release Profile. Polym. Bull..

[132] Ghanbarzadeh B., Almasi H., Entezami A.A. (2011). Improving the Barrier and Mechanical Properties of Corn Starch-Based Edible Films: Effect of Citric Acid and Carboxymethyl Cellulose. Ind. Crops Prod..

[133] Simões B.M., Cagnin C., Yamashita F., Olivato J.B., Garcia P.S., de Oliveira S.M., Eiras Grossmann M.V. (2020). Citric Acid as Crosslinking Agent in Starch/Xanthan Gum Hydrogels Produced by Extrusion and Thermopressing. Lwt.

[134] Yang J., Zhang X., Chen L., Zhou X., Fan X., Hu Y., Niu X., Xu X., Zhou G., Ullah N. (2022). Antibacterial Aerogels with Nano-silver Reduced in Situ by Carboxymethyl Cellulose for Fresh Meat Preservation. Int. J. Biol. Macromol..

[135] Künne S., Püttmann F., Linhorst M., Moerschbacher B.M., Winter M., Li J., Placke T. (2022). Comparative Study on Chitosans as Green Binder Materials for LiMn2O4 Positive Electrodes in Lithium Ion Batteries. ChemElectroChem.

[136] Tomadoni B., Ponce A., Pereda M., Ansorena M.R. (2019). Vanillin as a Natural Cross-Linking Agent in Chitosan-Based Films: Optimizing Formulation by Response Surface Methodology. Polym. Test..

[137] Brito G.B., Peixoto V.O.D.S., Martins M.T., Rosário D.K.A., Ract J.N., Conte-Júnior C.A., Torres A.G., Castelo-Branco V.N. (2022). Development of Chitosan-Based Oleogels via Crosslinking with Vanillin Using an Emulsion Templated Approach: Structural Characterization and Their Application as Fat-Replacer. Food Struct..

[138] Xie M., Hu B., Wang Y., Zeng X. (2014). Grafting of Gallic Acid onto Chitosan Enhances Antioxidant Activities and Alters Rheological Properties of the Copolymer. Agric. Food Chem..

[139] Khan M., Koivisto J.T., Kellomäki M., Kellomaki M., And B. (2022). Injectable and Self-Healing Biobased Composite Hydrogels as Future Anticancer Therapeutic Biomaterials. Nano Sel..

[140] Haute G.V., Caberlon E., Squizani E., de Mesquita F.C., Pedrazza L., Martha B.A., da Silva Melo D.A., Cassel E., Czepielewski R.S., Bitencourt S. (2015). Gallic Acid Reduces the Effect of LPS on Apoptosis and Inhibits the Formation of Neutrophil Extracellular Traps. Toxicol. In Vitro.

[141] Noel A., Borguet Y.P., Raymond J.E., Wooley K.L. (2014). Poly(Carbonate−amide)s Derived from Bio-Based Resources: Poly(Ferulic Acid-Co-Tyrosine). Macromolecules.

[142] Saletti M., Paolino M., Ballerini L., Giuliani G., Leone G., Lamponi S., Andreassi M., Bonechi C., Donati A., Piovani D. (2022). Click-Chemistry Cross-Linking of Hyaluronan Graft Copolymers. Pharmaceutics.

[143] Ouimet M.A., Griffin J., Carbone-Howell A.L., Wu W.H., Stebbins N.D., Di R., Uhrich K.E. (2013). Biodegradable Ferulic Acid-Containing Poly(Anhydride-Ester): Degradation Products with Controlled Release and Sustained Antioxidant Activity. Biomacromolecules.

[144] Cassimjee H., Kumar P., Ubanako P., Choonara Y.E. (2022). Genipin-Crosslinked, Proteosaccharide Scaffolds for Potential Neural Tissue Engineering Applications. Pharmaceutics.

[145] Du J.R., Hsu L.H., Xiao E.S., Guo X., Zhang Y., Feng X. (2020). Using Genipin as a “Green” Crosslinker to Fabricate Chitosan Membranes for Pervaporative Dehydration of Isopropanol. Sep. Purif. Technol..

[146] Bhattacharjee P., Ahearne M. (2021). Significance of Crosslinking Approaches in the Development of next Generation Hydrogels for Corneal Tissue Engineering. Pharmaceutics.

[147] Pal K., Paulson A.T., Rousseau D., Ebnesajjad S. (2013). Biopolymers in Controlled-Release Delivery Systems. Handbook of Biopolymers and Biodegradable Plastics.

[148] Pich A., Richtering W. (2012). Polymer Nanogels and Microgels. Polymer Science: A Comprehensive Reference.

[149] Kari F.W. (1993). National Toxicology Program Toxicity Report Series Number 25 NTP Technical Report on Toxicity Studies of Glutaraldehyde Administered by Inhalation to F344/N Rats and B6C3F 1 Mice. Toxic Rep. Ser..

[150] Mohamed R.R., Fahim M.E., Soliman S.M.A. (2022). Development of Hydrogel Based on Carboxymethyl Cellulose/Poly (4—Vinylpyridine) for Controlled Releasing of Fertilizers. BMC Chem..

[151] Serhan M., Sprowls M., Jackemeyer D., Long M., Perez I.D., Maret W., Tao N., Forzani E. (2012). Total Iron Measurement in Human Serum with a Smartphone. R. Soc. Chem..

[152] Mohana Raju K., Padmanabha Raju M., Murali Mohan Y. (2002). Synthesis and Water Absorbency of Crosslinked Superabsorbent Polymers. J. Appl. Polym. Sci..

[153] Kono H., Nakamura T. (2013). Polymerization of β-Cyclodextrin with 1,2,3,4-Butanetetracarboxylic Dianhydride: Synthesis, Structural Characterization, and Bisphenol A Adsorption Capacity. React. Funct. Polym..

[154] Zheng P., Lin Q., Li F., Ou Y., Chen N. (2017). Development and Characterization of a Defatted Soy Flour-Based Bio-Adhesive Crosslinked by 1,2,3,4-Butanetetracarboxylic Acid. Int. J. Adhes. Adhes..

[155] Lam Y., Kan C., Yuen C. (2011). Wrinkle-Resistant Finishing of Cotton Fabric with BTCA—The Effect of Co-Catalyst. Text. Res. J..

[156] Portocarrero Huang G., Shanmugasundaram S., Masih P., Pandya D., Amara S., Collins G., Livingston Arinzeh T. (2015). An Investigation of Common Crosslinking Agents on the Stability of Electrospun Collagen Scaffolds. J. Biomed. Mater. Res..

[157] Yeh M.K., Liang Y.M., Cheng K.M., Dai N.T., Liu C.C., Young J.J. (2011). A Novel Cell Support Membrane for Skin Tissue Engineering: Gelatin Film Cross-Linked with 2-Chloro-1-Methylpyridinium Iodide. Polymer.

[158] Zhang Y., Shi X., Yu Y., Zhao S., Song H., Chen A., Shang Z. (2014). Preparation and Characterization of Vanillin Cross-Linked Chitosan Microspheres of Pterostilbene. Int. J. Polym. Anal. Charact..

[159] Ismael M.N.M., El Nemr A., El Ashry E.S.H., Abdel Hamid H. (2020). Removal of Hexavalent Chromium by Cross-Linking Chitosan and N,N′-Methylene Bis-Acrylamide. Environ. Process..

[160] Shen S., Chen X., Shen Z., Chen H. (2021). Pharmaceutics Marine Polysaccharides for Wound Dressings Application: An Overview. Pharmaceutics.

[161] Orhan B., Kaygusuz H., Erim F.B. (2022). Sustainable Alginate-Carboxymethyl Cellulose Superabsorbents Prepared by a Novel Quasi-Cryogelation Method. J. Polym. Res..

[162] Zhao X., Lang Q., Yildirimer L., Lin Z.Y., Cui W., Annabi N., Ng K.W., Dokmeci M.R., Ghaemmaghami A.M., Khademhosseini A. (2016). Photocrosslinkable Gelatin Hydrogel for Epidermal Tissue Engineering. Adv. Healthc. Mater..

[163] Meftahi A., Khajavi R., Rashidi A., Rahimi M.K., Bahador A. (2018). Preventing the Collapse of 3D Bacterial Cellulose Network via Citric Acid. J. Nanostruct. Chem..

[164] Poole L.G., Kopec A.K., Flick M.J., Luyendyk J.P., Liaw P., James Luyendyk C.P. (2022). Cross-Linking by Tissue Transglutaminase-2 Alters Fibrinogen-Directed Macrophage Proinflammatory Activity. J. Thromb. Haemost..

[165] Matveeva V.G., Senokosova E.A., Sevostianova V.V., Khanova M.Y., Glushkova T.V., Akentieva T.N., Antonova L.V., Barbarash L.S. (2022). Advantages of Fibrin Polymerization Method without the Use of Exogenous Thrombin for Vascular Tissue Engineering Applications. Biomedicines.

[166] Nguyen T.X.Q., Chen S.S., Chang H.M., Cao N.D.T., Singh R. (2020). Effects of Polyethylene Glycol and Glutaraldehyde Cross-Linker on TFC-FO Membrane Performance. Environ. Technol. Innov..

[167] Nascimento F.C.D., de Aguiar L.C.V., Costa L.A.T., Fernandes M.T., Marassi R.J., Gomes A.D.S., de Castro J.A. (2021). Formulation and Characterization of Crosslinked Polyvinyl Alcohol (PVA) Membranes: Effects of the Crosslinking Agents. Polym. Bull..

[168] Ullah F., Othman M.B.H., Javed F., Ahmad Z., Akil H.M. (2015). Classification, Processing and Application of Hydrogels: A Review. Mater. Sci. Eng. C.

[169] Jyoti Bharali D., Maitra A., Mitra S. (2014). Hydrogel Nanoparticles: Cross-Linked Polyvinylpyrrolidone. Dekker Encyclopedia of Nanoscience and Nanotechnology.

[170] Jeon J.G., Kim H.C., Palem R.R., Kim J., Kang T.J. (2019). Cross-Linking of Cellulose Nanofiber Films with Glutaraldehyde for Improved Mechanical Properties. Mater. Lett..

[171] Liang L., Rieke P.C., Liu J., Fryxell G.E., Young J.S., Engelhard M.H., Alford K.L. (2000). Surfaces with Reversible Hydrophilic/Hydrophobic Characteristics on Cross-Linked Poly(N-Isopropylacrylamide) Hydrogels. Langmuir.

[172] Ullah Khan K., Akhtar N., Usman Minhas M. (2020). Poloxamer-407-Co-Poly (2-Acrylamido-2-Methylpropane Sulfonic Acid) Cross-Linked Nanogels for Solubility Enhancement of Olanzapine: Synthesis, Characterization, and Toxicity Evaluation. AAPS.

[173] Alshaikh R.A., Waeber C., Ryan K.B. (2022). Polymer Based Sustained Drug Delivery to the Ocular Posterior Segment: Barriers and Future Opportunities for the Treatment of Neovascular Pathologies. Adv. Drug Deliv. Rev..

[174] Wang M., Guo L., Sun H. (2019). Manufacture of Biomaterials. Encyclopedia of Biomedical Engineering.

[175] Badali E., Hosseini M., Mohajer M., Hassanzadeh S., Saghati S., Hilborn J., Khanmohammadi M. (2021). Enzymatic Crosslinked Hydrogels for Biomedical Application. Polym. Sci. Ser. A.

[176] Silvestro I., Francolini I., Di Lisio V., Martinelli A., Pietrelli L., Scotto d’Abusco A., Scoppio A., Piozzi A. (2020). Preparation and Characterization of TPP-Chitosan Crosslinked Scaffolds for Tissue Engineering. Materials.

[177] Cruz A., Couto L., Esplugas S., Sans C. (2017). Study of the Contribution of Homogeneous Catalysis on Heterogeneous Fe(III)/Alginate Mediated Photo-Fenton Process. Chem. Eng. J..

[178] Wahab A.H.A., Saad A.P.M., Harun M.N., Syahrom A., Ramlee M.H., Sulong M.A., Kadir M.R.A. (2019). Developing Functionally Graded PVA Hydrogel Using Simple Freeze-Thaw Method for Artificial Glenoid Labrum. J. Mech. Behav. Biomed. Mater..

[179] Pearce H.A., Kim Y.S., Diaz-Gomez L., Mikos A.G. (2020). Tissue Engineering Scaffolds. Biomater. Sci..

[180] Kirtania M.D., Kahali N., Maity A. (2021). Inulin-Based Hydrogel. Plant and Algal Hydrogels for Drug Delivery and Regenerative Medicine.

[181] Li Y., Wang X., Han Y., Sun H.Y., Hilborn J., Shi L. (2021). Click Chemistry-Based Biopolymeric Hydrogels for Regenerative Medicine. Biomed. Mater..

[182] Mohabatpour F., Yazdanpanah Z., Papagerakis S., Chen X., Papagerakis P. (2022). Self-Crosslinkable Oxidized Alginate-Carboxymethyl Chitosan Hydrogels as an Injectable Cell Carrier for In Vitro Dental Enamel Regeneration. J. Funct. Biomater..

[183] Astudillo-Ortiz E., Babo P.S., Reis R.L., Gomes M.E. (2021). Evaluation of Injectable Hyaluronic Acid-Based Hydrogels for Endodontic Tissue Regeneration. Materials.

[184] Sellers D.L., Kim T.H., Mount C.W., Pun S.H., Horner P.J. (2014). Poly(Lactic-Co-Glycolic) Acid Microspheres Encapsulated in Pluronic F-127 Prolong Hirudin Delivery and Improve Functional Recovery from a Demyelination Lesion. Biomaterials.

[185] Nguyen L.H., Gao M., Lin J., Wu W., Wang J., Chew S.Y. (2017). Three-Dimensional Aligned Nanofibers-Hydrogel Scaffold for Controlled Non-Viral Drug/Gene Delivery to Direct Axon Regeneration in Spinal Cord Injury Treatment. Sci. Rep..

[186] Calias P., Banks W.A., Begley D., Scarpa M., Dickson P. (2014). Intrathecal Delivery of Protein Therapeutics to the Brain: A Critical Reassessment. Pharmacol. Ther..

[187] Fowler M.J., Cotter J.D., Knight B.E., Sevick-Muraca E.M., Sandberg D.I., Sirianni R.W. (2020). Intrathecal Drug Delivery in the Era of Nanomedicine. Adv. Drug Deliv. Rev..

[188] LeBel C., Bourdeau A., Lau D., Hunt P. (1999). Biologic Response to Peripheral and Central Administration of Recombinant Human Leptin in Dogs. Obes. Res..

[189] Yousfan A., Rubio N., Natouf A.H., Daher A., Al-Kafry N., Venner K., Kafa H. (2020). Preparation and Characterization of PHT-Loaded Chitosan Lecithin Nanoparticles for Intranasal Drug Delivery to the Brain. RSC Adv..

[190] Keller L.-A., Merkel O., Popp A. (2022). Intranasal Drug Delivery: Opportunities and Toxicologic Challenges during Drug Development. Drug Deliv. Transl. Res..

[191] Liu S., Yang S., Ho P.C. (2018). Intranasal Administration of Carbamazepine-Loaded Carboxymethyl Chitosan Nanoparticles for Drug Delivery to the Brain. Asian J. Pharm. Sci..

[192] Melamane S., Walker R.B., Khamanga S.M.M. (2020). Formulation Optimization of Smart Thermosetting Lamotrigine Loaded Hydrogels Using Response Surface Methodology, Box Benhken Design and Artificial Neural Networks. Drug Dev. Ind. Pharm..

[193] Karna S. (2020). Commentary: Eye as a Window to the Brain. Indian J. Ophthalmol..

[194] Majeed A., Khan N.A. (2019). Ocular in Situ Gel: An Overview. J. Drug Deliv. Ther..

[195] Gupta S. (2010). Carbopol/Chitosan Based PH Triggered In Situ Gelling System for Ocular Delivery of Timolol Maleate. Sci. Pharm..

[196] Dai M., Bai L., Zhang H., Ma Q., Luo R., Lei F., Fei Q., He N. (2020). A Novel Flunarizine Hydrochloride-Loaded Organogel for Intraocular Drug Delivery in Situ: Design, Physicochemical Characteristics and Inspection. Int. J. Pharm..

[197] Vashist A., Kaushik A., Ghosal A., Bala J., Nikkhah-Moshaie R., Wani W.A., Manickam P., Nair M. (2018). Nanocomposite Hydrogels: Advances in Nanofillers Used for Nanomedicine. Gels.

[198] Merino S., Martín C., Kostarelos K., Prato M., Vázquez E. (2015). Nanocomposite Hydrogels: 3D Polymer-Nanoparticle Synergies for on-Demand Drug Delivery. ACS Nano.

[199] Bhat A.H., Khan I., Amil Usmani M., Rather J.A. (2016). Bioplastics and Bionanocomposites Based on Nanoclays and Other Nanofillers. Nanoclay Reinforced Polymer Composites.

[200] Wu C., Liu J., Zhai Z., Yang L., Tang X., Zhao L., Xu K., Zhong W. (2020). Double-Crosslinked Nanocomposite Hydrogels for Temporal Control of Drug Dosing in Combination Therapy. Acta Biomater..

[201] Liu Y., Meng H., Qian Z., Fan N., Choi W., Zhao F., Lee B.P. (2017). A Moldable Nanocomposite Hydrogel Composed of a Mussel-Inspired Polymer and a Nanosilicate as a Fit-to-Shape Tissue Sealant. Angew. Chem. Int. Ed..

[202] Gong C., Lu C., Li B., Shan M., Wu G. (2017). Injectable Dopamine-Modified Poly(α,β-Aspartic Acid) Nanocomposite Hydrogel as Bioadhesive Drug Delivery System. J. Biomed. Mater. Res. Part A.

[203] Zhao F., Yao D., Guo R., Deng L., Dong A., Zhang J. (2015). Composites of Polymer Hydrogels and Nanoparticulate Systems for Biomedical and Pharmaceutical Applications. Nanomaterials.

[204] Tao J., Zhang J., Hu Y., Yang Y., Gou Z., Du T., Mao J., Gou M. (2017). A Conformal Hydrogel Nanocomposite for Local Delivery of Paclitaxel. J. Biomater. Sci. Polym. Ed..

[205] Jiang Y., Chen J., Deng C., Suuronen E.J., Zhong Z. (2014). Click Hydrogels, Microgels and Nanogels: Emerging Platforms for Drug Delivery and Tissue Engineering. Biomaterials.

[206] Yadav H.K., Halabi A., Alsalloum N.A. Nanogels as Novel Drug Delivery Systems—A Review. https://www.semanticscholar.org/paper/Nanogels-as-Novel-Drug-Delivery-Systems-A-Review-Yadav-Halabi/89312e50c1c0c1dd83d81669295508cce5e7c370.

[207] Jain S., Ancheria R.K., Shrivastava S., Soni S.L., Sharma M. (2019). An Overview of Nanogel–Novel Drug Delivery System. Asian J. Pharm. Res. Dev..

[208] Anooj E., Charumathy M., Sharma V., Vibala B.V., Gopukumar S.T., Jainab S.I.B., Vallinayagam S. (2021). Nanogels: An Overview of Properties, Biomedical Applications, Future Research Trends and Developments. J. Mol. Struct..

[209] Ahmed S., Alhareth K., Mignet N. (2020). Advancement in Nanogel Formulations Provides Controlled Drug Release. Int. J. Pharm..

[210] Hajebi S., Rabiee N., Bagherzadeh M., Ahmadi S., Rabiee M., Roghani-Mamaqani H., Tahriri M., Tayebi L., Hamblin M.R. (2019). Stimulus-Responsive Polymeric Nanogels as Smart Drug Delivery Systems. Acta Biomater..

[211] Vinogradov S.V., Batrakova E.V., Kabanov A.V. (2004). Nanogels for Oligonucleotide Delivery to the Brain. Bioconjug. Chem..

[212] Li Q., Shao X., Dai X., Guo Q., Yuan B., Liu Y., Jiang W. (2022). Recent Trends in the Development of Hydrogel Therapeutics for the Treatment of Central Nervous System Disorders. NPG Asia Mater..

[213] Upadhyay R.K. (2014). Drug Delivery Systems, CNS Protection, and the Blood Brain Barrier. Biomed Res. Int..

[214] Vashist A., Kaushik A., Vashist A., Bala J., Nikkhah-Moshaie R., Sagar V., Nair M. (2018). Nanogels as Potential Drug Nanocarriers for CNS Drug Delivery. Drug Discov. Today.

[215] Sun H., Zhang L., Cheng W., Hao F., Zhou L., Li Q. (2021). Injectable Hydrogels in Repairing Central Nervous System Injuries. Adv. Mater. Sci. Eng..

[216] Correll C.U., Kim E., Sliwa J.K., Hamm W., Gopal S., Mathews M., Venkatasubramanian R., Saklad S.R. (2021). Pharmacokinetic Characteristics of Long-Acting Injectable Antipsychotics for Schizophrenia: An Overview. CNS Drugs.

[217] Tobinick E.L. (2016). Perispinal Delivery of CNS Drugs. CNS Drugs.

[218] Shatsberg Z., Zhang X., Ofek P., Malhotra S., Krivitsky A., Scomparin A., Tiram G., Calderón M., Haag R., Satchi-Fainaro R. (2016). Functionalized Nanogels Carrying an Anticancer MicroRNA for Glioblastoma Therapy. J. Control. Release.

[219] She D., Huang H., Li J., Peng S., Wang H., Yu X. (2021). Hypoxia-Degradable Zwitterionic Phosphorylcholine Drug Nanogel for Enhanced Drug Delivery to Glioblastoma. Chem. Eng. J..

[220] Jiang L., Zhou Q., Mu K., Xie H., Zhu Y., Zhu W., Zhao Y., Xu H., Yang X. (2013). PH/Temperature Sensitive Magnetic Nanogels Conjugated with Cy5.5-Labled Lactoferrin for MR and Fluorescence Imaging of Glioma in Rats. Biomaterials.

[221] Nakamura H. (2020). Development of Tumor-Targeting Antitumor Agents Based on Polymer Effect. Yakugaku Zasshi J. Pharm. Soc. Jpn..

[222] Qian F., Han Y., Han Z., Zhang D., Zhang L., Zhao G., Li S., Jin G., Yu R., Liu H. (2021). In Situ Implantable, Post-Trauma Microenvironment-Responsive, ROS Depletion Hydrogels for the Treatment of Traumatic Brain Injury. Biomaterials.

[223] Nazemi Z., Nourbakhsh M.S., Kiani S., Heydari Y., Ashtiani M.K., Daemi H., Baharvand H. (2020). Co-Delivery of Minocycline and Paclitaxel from Injectable Hydrogel for Treatment of Spinal Cord Injury. J. Control. Release.

[224] Li J., Darabi M., Gu J., Shi J., Xue J., Huang L., Liu Y., Zhang L., Liu N., Zhong W. (2016). A Drug Delivery Hydrogel System Based on Activin B for Parkinson’s Disease. Biomaterials.

[225] Zhang K., Shi Z., Zhou J., Xing Q., Ma S., Li Q., Zhang Y., Yao M., Wang X., Li Q. (2018). Potential Application of an Injectable Hydrogel Scaffold Loaded with Mesenchymal Stem Cells for Treating Traumatic Brain Injury. J. Mater. Chem. B.

[226] Kang N.-W., Yoon S.-Y., Kim S., Yu N.-Y., Park J.-H., Lee J.-Y., Cho H.-J., Kim D.-D. (2021). Subcutaneously Injectable Hyaluronic Acid Hydrogel for Sustained Release of Donepezil with Reduced Initial Burst Release: Effect of Hybridization of Microstructured Lipid Carriers and Albumin. Pharmaceutics.

[227] Gerson T., Makarov E., Senanayake T.H., Gorantla S., Poluektova L.Y., Vinogradov S.V. (2014). Nano-NRTIs Demonstrate Low Neurotoxicity and High Antiviral Activity against HIV Infection in the Brain. Nanomed. Nanotechnol. Biol. Med..

[228] Jahromi L.P., Mohammadi-samani S., Heidari R., Azadi A., Sciences P. (2018). In Vitro- and in Vivo Evaluation of Methotrexate-Loaded Hydrogel. J. Pharm. Pharm. Sci..

[229] Baklaushev V.P., Nukolova N.N., Khalansky A.S., Gurina O.I., Yusubalieva G.M., Grinenko N.P., Gubskiy I.L., Melnikov P.A., Kardashova K.S., Kabanov A.V. (2015). Treatment of Glioma by Cisplatin-Loaded Nanogels Conjugated with Monoclonal Antibodies against Cx43 and BSAT1. Drug Deliv..

[230] Ikeda K., Okada T., Sawada S., Akiyoshi K., Matsuzaki K. (2006). Inhibition of the Formation of Amyloid β-Protein Fibrils Using Biocompatible Nanogels as Artificial Chaperones. FEBS Lett..

[231] Wang Y., Ying X., Chen L., Liu Y., Wang Y., Liang J., Xu C., Guo Y., Wang S., Hu W. (2016). Electroresponsive Nanoparticles Improve Antiseizure Effect of Phenytoin in Generalized Tonic-Clonic Seizures. Neurotherapeutics.

[232] Qi X.-J., Xu D., Tian M.-L., Zhou J.-F., Wang Q.-S., Cui Y.-L. (2021). Thermosensitive Hydrogel Designed for Improving the Antidepressant Activities of Genipin via Intranasal Delivery. Mater. Des..

[233] Chen W., Zou Y., Zhong Z., Haag R. (2017). Cyclo(RGD)-Decorated Reduction-Responsive Nanogels Mediate Targeted Chemotherapy of Integrin Overexpressing Human Glioblastoma In Vivo. Small.

[234] Azadi A., Hamidi M., Rouini M.-R. (2013). Methotrexate-Loaded Chitosan Nanogels as ‘Trojan Horses’ for Drug Delivery to Brain: Preparation and in Vitro/in Vivo Characterization. Int. J. Biol. Macromol..

[235] Galgatte U.C., Kumbhar A.B., Chaudhari P.D. (2014). Development of in Situ Gel for Nasal Delivery: Design, Optimization, in Vitro and in Vivo Evaluation. Drug Deliv..

[236] Qian S., Wong Y.C., Zuo Z. (2014). Development, Characterization and Application of in Situ Gel Systems for Intranasal Delivery of Tacrine. Int. J. Pharm..

[237] Abdulla N.A., Balata G.F., El-ghamry H.A., Gomaa E. (2021). Intranasal Delivery of Clozapine Using Nanoemulsion-Based in-Situ Gels: An Approach for Bioavailability Enhancement. Saudi Pharm. J..

[238] Haidary H.A., Padhy R.K. Clozapine. http://www.ncbi.nlm.nih.gov/pubmed/30571020.

[239] McCrorie P., Mistry J., Taresco V., Lovato T., Fay M., Ward I., Ritchie A.A., Clarke P.A., Smith S.J., Marlow M. (2020). Etoposide and Olaparib Polymer-Coated Nanoparticles within a Bioadhesive Sprayable Hydrogel for Post-Surgical Localised Delivery to Brain Tumours. Eur. J. Pharm. Biopharm..

[240] Picone P., Sabatino M.A., Ditta L.A., Amato A., San Biagio P.L., Mulè F., Giacomazza D., Dispenza C., Di Carlo M. (2018). Nose-to-Brain Delivery of Insulin Enhanced by a Nanogel Carrier. J. Control. Release.

[241] Madhav S., Dewari A., Tyagi Y. (2020). Asian Journal of Nanoscience and Materials An Innovative Approach Delivery of Anticonvulsant via Transcranial Route Using a Smart Bio-Functional Agent Cum Musa Acuminata. Asian J. Nanosci. Mater..

[242] Salem H.F., Kharshoum R.M., Abou-Taleb H.A., Naguib D.M. (2019). Nanosized Nasal Emulgel of Resveratrol: Preparation, Optimization, in Vitro Evaluation and in Vivo Pharmacokinetic Study. Drug Dev. Ind. Pharm..

[243] Al Harthi S., Alavi S.E., Radwan M.A., El Khatib M.M., AlSarra I.A. (2019). Nasal Delivery of Donepezil HCl-Loaded Hydrogels for the Treatment of Alzheimer’s Disease. Sci. Rep..

